# *KRAS* mutant–driven SUMOylation controls extracellular vesicle transmission to trigger lymphangiogenesis in pancreatic cancer

**DOI:** 10.1172/JCI157644

**Published:** 2022-07-15

**Authors:** Yuming Luo, Zhihua Li, Yao Kong, Wang He, Hanhao Zheng, Mingjie An, Yan Lin, Dingwen Zhang, Jiabin Yang, Yue Zhao, Changhao Chen, Rufu Chen

**Affiliations:** 1 Department of Pancreatic Surgery, Guangdong Provincial People’s Hospital, Guangdong Academy of Medical Sciences, Guangzhou, China.; 2 Department of Oncology, Sun Yat-sen Memorial Hospital, Guangzhou, China.; 3 Guangdong Cardiovascular Institute, Guangzhou, China.; 4 Department of Urology, Sun Yat-sen Memorial Hospital, Sun Yat-sen University, Guangzhou, China.; 5 Guangdong Provincial Key Laboratory of Malignant Tumor Epigenetics and Gene Regulation, Sun Yat-sen Memorial Hospital, State Key Laboratory of Oncology in South China, Guangzhou, China.; 6 School of Medicine, South China University of Technology, Guangzhou, China.; 7 Department of Tumor Intervention, Sun Yat-sen University First Affiliated Hospital, Guangzhou, China.

**Keywords:** Oncology, Cancer, Lymph, Molecular biology

## Abstract

Lymph node (LN) metastasis occurs frequently in pancreatic ductal adenocarcinoma (PDAC) and predicts poor prognosis for patients. The *KRAS^G12D^* mutation confers an aggressive PDAC phenotype that is susceptible to lymphatic dissemination. However, the regulatory mechanism underlying *KRAS^G12D^* mutation–driven LN metastasis in PDAC remains unclear. Herein, we found that PDAC with the *KRAS^G12D^* mutation (*KRAS^G12D^* PDAC) sustained extracellular vesicle–mediated (EV-mediated) transmission of heterogeneous nuclear ribonucleoprotein A1 (hnRNPA1) in a SUMOylation-dependent manner and promoted lymphangiogenesis and LN metastasis in vitro and in vivo. Mechanistically, hnRNPA1 bound with SUMO2 at the lysine 113 residue via *KRAS^G12D^*-induced hyperactivation of SUMOylation, which enabled its interaction with TSG101 to enhance hnRNPA1 packaging and transmission via EVs. Subsequently, SUMOylation induced EV-packaged-hnRNPA1 anchoring to the adenylate- and uridylate-rich elements of *PROX1* in lymphatic endothelial cells, thus stabilizing *PROX1* mRNA. Importantly, impeding SUMOylation of EV-packaged hnRNPA1 dramatically inhibited LN metastasis of *KRAS^G12D^* PDAC in a genetically engineered *Kras^G12D/+^* *Trp53^R172H/+^* *Pdx-1*-Cre (KPC) mouse model. Our findings highlight the mechanism by which *KRAS* mutant–driven SUMOylation triggers EV-packaged hnRNPA1 transmission to promote lymphangiogenesis and LN metastasis, shedding light on the potential application of hnRNPA1 as a therapeutic target in patients with *KRAS^G12D^* PDAC.

## Graphical abstract

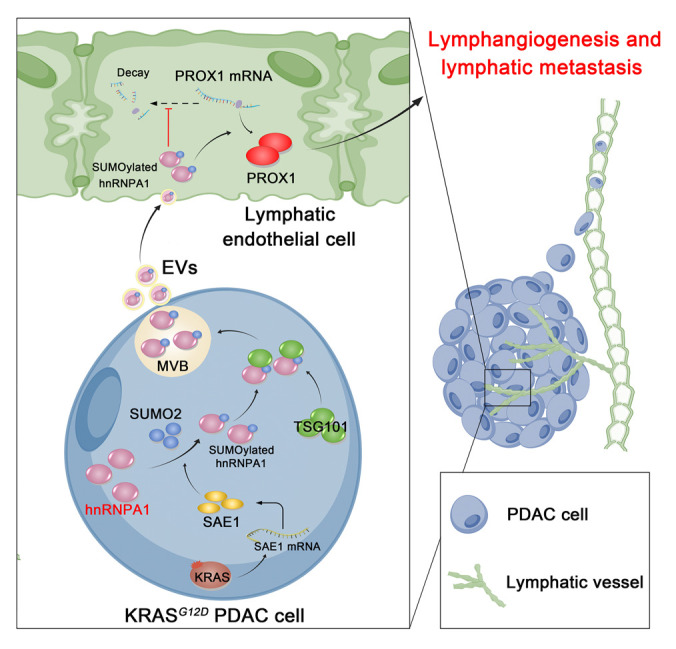

## Introduction

Pancreatic ductal adenocarcinoma (PDAC) is one of the most malignant digestive system cancers and represents the seventh leading cause of cancer-related death worldwide (1, 2). Accumulating reports have shown that lymph node (LN) metastasis represents the major metastatic route of PDAC and that it predicts extremely poor prognosis, where it decreases the 5-year survival rate of patients who have received pancreatoduodenectomy or distal pancreatectomy from 40% to 10% (3, 4). The development of LN metastasis in PDAC requires multiple complex processes, among which lymphangiogenesis, the generation and sprouting of lymphatic vessels from pre-existing lymphatic vasculature, represents the predominant step (5–8). The current antilymphangiogenesis therapies with monoclonal antibodies, micromolecular peptides, or inhibitors targeting vascular endothelial growth factor (VEGF) signaling, the well-characterized pathway for inducing lymphatic vasculature, have achieved limited efficacy against metastatic PDAC in the past decade, prompting the need for developing therapeutic targets of LN metastatic PDAC (3).

KRAS has been well characterized as a membrane-bound GTPase widely involved in cell growth, migration, and survival (9, 10). One-fifth of all human cancers, including 85%–90% of PDAC, harbor *KRAS* activating mutations (9). The *KRAS^G12D^* mutation is the most prevalent mutation among the PDAC-associated *KRAS* mutations, causing pancreatic duct epithelium transition to focal premalignant ductal lesions and also inducing rapid progression to highly invasive and metastatic PDAC by fostering the hyperactivation of several central cellular growth signaling pathways, including mitogen-activated protein kinase (MAPK), phosphatidylinositol 3-kinase (PI3K), and Ras-like GEF (RalGEF) (11, 12). *KRAS^G12D^* mutation–related activation promotes the aggregation of tumor cells around lymphatic vessels, which has been associated with the presence of LN metastasis in PDAC (13, 14). Nonetheless, the precise mechanism of *KRAS* mutation in PDAC lymphangiogenesis and LN metastasis remains unclear.

Extracellular vesicles (EVs), membrane-enclosed vesicles 30–150 nm in diameter, have been widely acknowledged as vital communication mediators during cancer development (15, 16). Tumor cell–secreted EVs play an important role in reshaping the tumor microenvironment (TME) by transferring biological molecules to modulate stromal cell metabolism and self-renewal, resulting in tumor metastasis (17, 18). The application of fibroblast-like mesenchymal cell–derived EVs for transmitting small interfering RNA (siRNA) specifically targeting the *KRAS^G12D^* mutation achieved satisfactory efficacy in inhibiting PDAC progression and now are undergoing phase I/II clinical testing (19). Therefore, elucidating the mechanism of EVs in *KRAS* mutant–triggered PDAC LN metastasis is of great clinical importance for developing the effective engineering of an EV-dependent therapeutic approach against LN metastatic PDAC.

In the present study, we demonstrated that the *KRAS^G12D^* mutation was accompanied by lymphangiogenesis hyperactivation in PDAC, and found that heterogeneous nuclear ribonucleoprotein A1 (hnRNPA1) was specifically upregulated in *KRAS^G12D^* PDAC cell–secreted EVs, which was positively associated with LN metastasis of *KRAS^G12D^* PDAC. HnRNPA1 packaged by *KRAS^G12D^* PDAC cell–secreted EVs was transmitted to human lymphatic endothelial cells (HLECs) to promote lymphangiogenesis and LN metastasis in vitro and in vivo. Moreover, hnRNPA1 was SUMOylated by *KRAS^G12D^* mutation–induced overexpression of SUMO-activating enzyme subunit 1 (SAE1), which triggered EV packaging of hnRNPA1 and its delivery to HLECs and subsequently facilitated *KRAS^G12D^* PDAC lymphangiogenesis and LN metastasis. Our results highlight a mechanism by which the *KRAS^G12D^* mutation induces lymphangiogenesis and LN metastasis by controlling SUMOylation-related transmission of EV-packaged hnRNPA1 in PDAC, highlighting the possibility that hnRNPA1 may be an attractive therapeutic target in *KRAS^G12D^* PDAC.

## Results

### HnRNPA1 is correlated with LN metastasis in KRAS^G12D^ PDAC.

**Figure 1:**
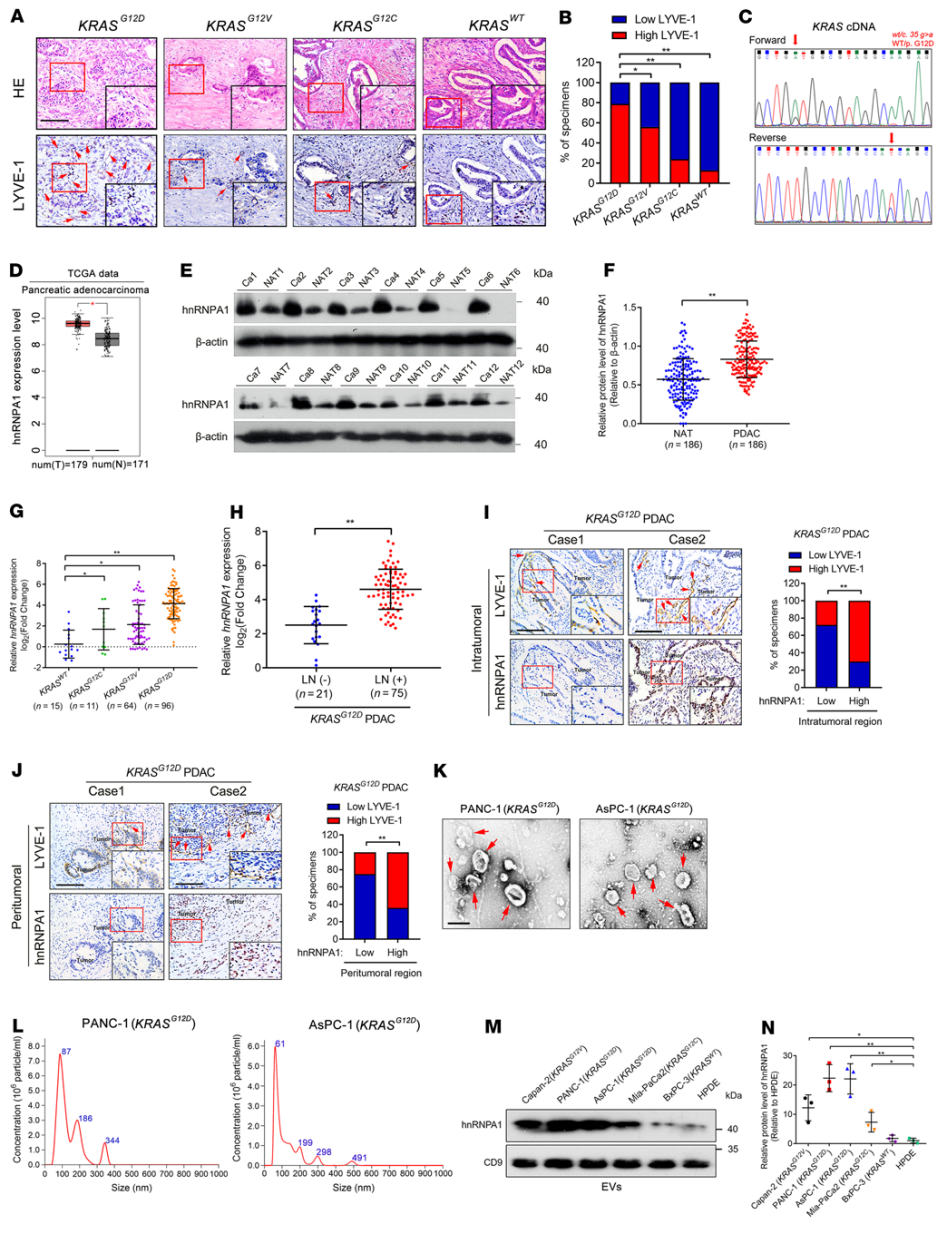
HnRNPA1 correlates with LN metastasis of *KRAS^G12D^* PDAC. (**A** and **B**) Representative H&E-stained and IHC images (**A**) and percentages of LYVE-1–positive lymphatic vessel density (**B**) in PDAC according to *KRAS* subtype (*KRAS^WT^*, *n =* 15; *KRAS^G12C^*, *n =* 11; *KRAS^G12V^*, *n =* 64; *KRAS^G12D^*, *n =* 96). Scale bars: 50 μm (black) or 25 μm (red). The χ^2^ test was used. (**C**) Sequencing evaluation of the *KRAS^G12D^* mutation. (**D**) HnRNPA1 expression in PDAC and normal pancreatic tissues was analyzed using data from TCGA database. The nonparametric Mann-Whitney *U* test was used. (**E** and **F**) Representative Western blotting images and quantification of hnRNPA1 expression in PDAC tissues and paired normal adjacent tissue (NAT) (*n =* 186). The nonparametric Mann-Whitney *U* test was used. (**G**) qRT-PCR of *hnRNPA1* expression in PDAC tissues (*n =* 186) according to *KRAS* subtype. The nonparametric Mann-Whitney *U* test was used. (**H**) qRT-PCR of *hnRNPA1* expression in LN-positive and LN-negative *KRAS^G12D^* PDAC tissues (*n =* 186). The nonparametric Mann-Whitney *U* test was used. (**I** and **J**) Representative images and percentages of IHC staining for hnRNPA1 expression and LYVE-1–positive lymphatic vessel density in *KRAS^G12D^* PDAC. Scale bars: 50 μm. The χ^2^ test was used. (**K** and **L**) TEM- (**K**) and NanoSight-characterized (**L**) EVs secreted by *KRAS^G12D^* PDAC cells. Scale bar: 100 nm. (**M** and **N**) Western blotting images and quantification of hnRNPA1 expression in EVs secreted by PDAC cells with different *KRAS* subtypes and HPDE cells. One-way ANOVA followed by Dunnett’s test was used. Data are presented as mean ± SD; 3 independent experiments were performed in **K**–**N**. The box-and-whisker plot in **D** represents medians with minimum and maximum values. The top and bottom of the box represent the first and third quartiles. *P < 0.05, **P < 0.01.

*KRAS^G12D^* represents the leading mutation in PDAC and causes tumor cell aggregation around lymphatic vessels, implying that it might be related to tumor metastasis through lymphatic vasculature in PDAC (13). Therefore, the *KRAS* mutations in our clinical PDAC samples were verified in-house by Sanger sequencing, and analysis of the samples by immunohistochemistry (IHC) showed an increase in microlymphatic vessels in the *KRAS^G12D^* PDAC tissues as compared with cancer tissues with other *KRAS* subtypes (Figure 1, A–C). As lymphatic vessel expansion is conducive to tumor cell metastasis to the LNs, we analyzed the correlation between *KRAS^G12D^* and LN metastasis of PDAC. A higher rate of LN metastasis was observed in PDAC with *KRAS^G12D^* mutation than in PDAC with other *KRAS* subtypes, suggesting that the *KRAS^G12D^* mutation was associated with LN metastasis of PDAC (Supplemental Figure 1A; supplemental material available online with this article; https://doi.org/10.1172/JCI157644DS1). Given that we and others have revealed that the majority of cancer-associated RNAs trigger tumor lymphangiogenesis by interacting with RNA-binding proteins (RBPs), among which hnRNPs were previously demonstrated to be the specific type of RBPs that correlated with various tumor LN metastasis (5, 20), we investigated the hnRNPs that contributed to *KRAS^G12D^*-associated lymphangiogenesis and LN metastasis in PDAC. First, the screening of hnRNPs was performed in PDAC and nontumorous tissues from The Cancer Genome Atlas (TCGA) and Genotype-Tissue Expression (GTEx) databases, which showed that 3 hnRNPs, including hnRNPA1, RALY, and SYNCRIP, were upregulated in PDAC versus nontumorous tissues by more than 2-fold and were correlated with poor prognosis of patients with PDAC (Figure 1D, Supplemental Figure 1, B–M, and Supplemental Table 1). Further validation in a larger cohort of 186 cases of PDAC patients by both quantitative reverse transcription PCR (qRT-PCR) and Western blotting analysis showed that hnRNPA1 was significantly overexpressed in PDAC and correlated with the *KRAS^G12D^* mutation (Figure 1, E–G, Supplemental Figure 1N, and Supplemental Figure 2). Kaplan-Meier curve analysis demonstrated that hnRNPA1 overexpression was associated with shorter overall survival (OS) and disease-free survival (DFS) of patients with *KRAS^G12D^* PDAC (Supplemental Figure 3, A and B), indicating that hnRNPA1 is a crucial participant in *KRAS^G12D^* PDAC. Accordingly, hnRNPA1 was selected for further analysis.

Clinical relevance analysis revealed that hnRNPA1 was overexpressed in patients with *KRAS^G12D^* PDAC with LN metastasis as compared with those without LN metastasis (Figure 1H and Supplemental Figure 3C). Moreover, we observed a positive correlation between hnRNPA1 expression and microlymphatic vessel density indicated by lymphatic vessel endothelial hyaluronan receptor 1 (LYVE-1) in both the intratumoral and peritumoral regions of *KRAS^G12D^* PDAC tissues (Figure 1, I and J), indicating that hnRNPA1 is correlated with lymphangiogenesis in *KRAS^G12D^* PDAC. Taken together, these findings reveal that hnRNPA1 is associated with lymphangiogenesis and LN metastasis of *KRAS^G12D^* PDAC.

### HnRNPA1 is enriched in EVs secreted by KRAS^G12D^ PDAC cells.

Strikingly, we found that hnRNPA1 existed in the extracellular region of *KRAS^G12D^* PDAC tissues (Supplemental Figure 3C). The *KRAS^G12D^* PDAC tissues with LN metastasis had higher extracellular hnRNPA1 expression than those without LN metastasis (Supplemental Figure 3C), indicating that hnRNPA1 might facilitate *KRAS^G12D^* PDAC LN metastasis in its extracellular form. Given that EVs, the nanoscale carriers for communication between tumor cells and stromal cells, have been considered to mediate molecules crossing the extracellular matrix into lymphatic circulation (21), we isolated the EVs from the culture media of PDAC cells with different *KRAS* subtypes (*KRAS^G12D^*: PANC-1, AsPC-1; *KRAS^G12V^*: Capan-2; *KRAS^G12C^*: Mia-PaCa-2; *KRAS^WT^*: BxPC-3) to investigate whether hnRNPA1 exhibited its function in *KRAS^G12D^* PDAC cell–secreted EVs. Transmission electron microscopy (TEM) and nanoparticle tracking analysis (NTA) identified cup-shaped particles 50 to 130 nm in size (Figure 1, K and L, and Supplemental Figure 3, D and E). Western blotting analysis revealed a higher expression level of the EV markers ALG-2–interacting protein X (ALIX), CD63, and CD9 in the isolated particles than the cellular lysate, while the cellular marker calnexin was rarely detected in the isolated particles (Supplemental Figure 3F), supporting the idea that the isolated particles were EVs. HnRNPA1 was specifically upregulated in *KRAS^G12D^* PDAC cells and the corresponding EVs as compared with PDAC cells with other *KRAS* subtypes or normal human pancreatic ductal epithelial (HPDE) cells (Figure 1, M and N, and Supplemental Figure 4, A and B). Since the TME of PDAC is accompanied with highly infiltrated cells, which release abundant EVs into the extracellular space of PDAC tissues, we also evaluated the expression of hnRNPA1 in EVs secreted by the predominant cells in the TME of PDAC, including fibroblasts, macrophages, T cells, and B cells, as well as the tumor cells. The results showed that hnRNPA1 expression was significantly higher in EVs from *KRAS^G12D^* PDAC cells compared with EVs secreted by the other cells in the TME (Supplemental Figure 4, C and D), indicating that hnRNPA1 is predominantly enriched in EVs secreted by *KRAS^G12D^* PDAC cells.

### EV-packaged hnRNPA1 secreted by KRAS^G12D^ PDAC cells enhances tube formation and migration of HLECs in vitro.

**Figure 2:**
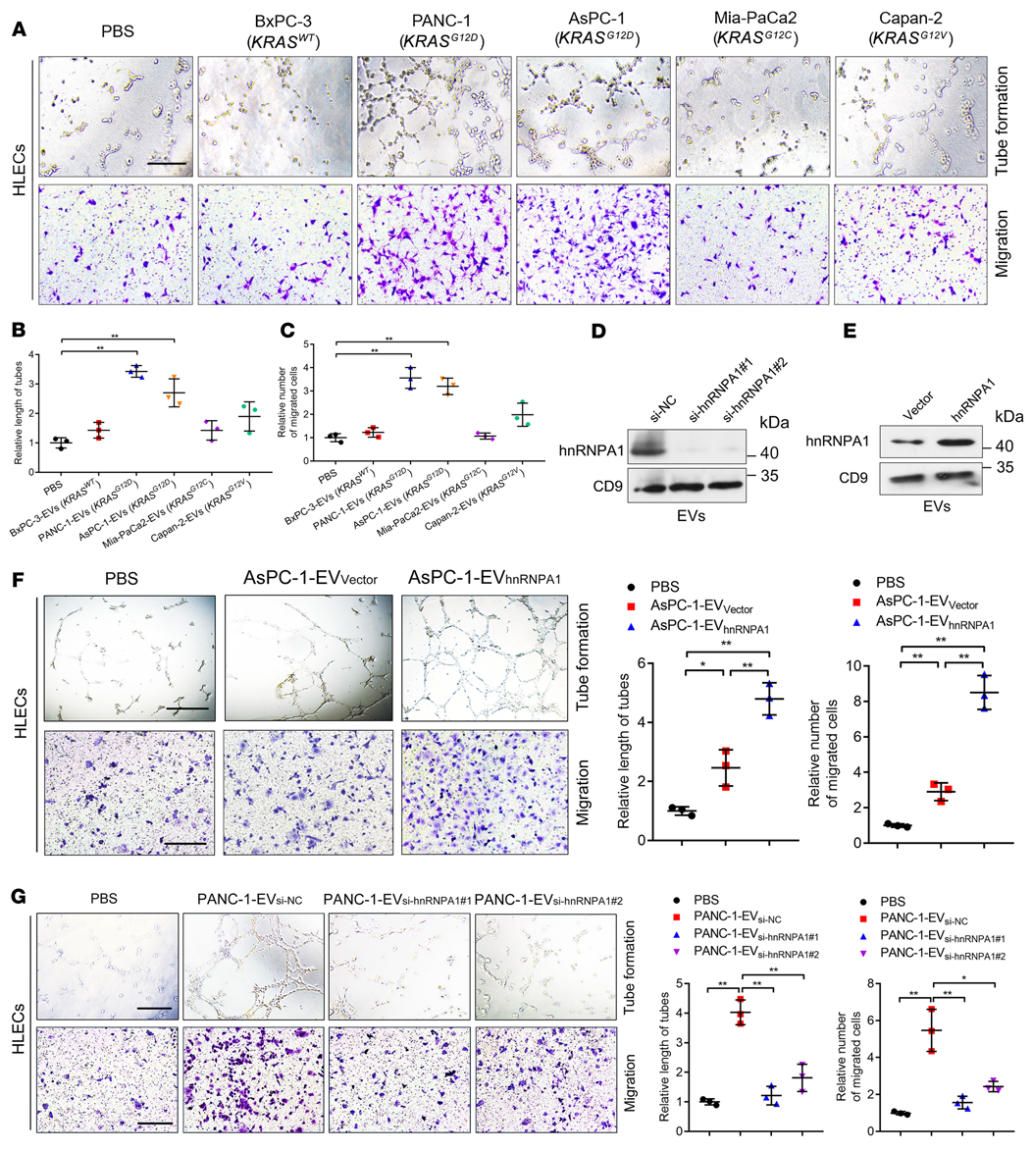
EV-packaged hnRNPA1 promotes lymphangiogenesis in vitro. (**A**–**C**) Representative images (**A**) and quantification of tube formation and migration (**B** and **C**) for HLECs treated with PBS or PDAC cell–secreted EVs. Scale bar: 100 μm. One-way ANOVA followed by Dunnett’s test was used. (**D** and **E**) Western blotting analysis of hnRNPA1 protein levels in PANC-1 cell–secreted EVs after hnRNPA1 silencing or overexpression. (**F** and **G**) Representative images and quantification of tube formation and migration by HLECs treated with PBS or indicated EVs. Scale bars: 100 μm. One-way ANOVA followed by Dunnett’s test was used. Data are presented as mean ± SD of 3 independent experiments. *P < 0.05, **P < 0.01.

Considering that lymphangiogenesis represents the determinant process mediating lymphatic dissemination of PDAC cells to the draining LNs and fosters LN metastasis, we explored the role of EV-packaged hnRNPA1 in the tube formation and migration of HLECs in vitro. EVs secreted by PANC-1 and ASPC-1 (*KRAS^G12D^*) cells with higher hnRNPA1 expression levels markedly promoted HLEC tube formation and migration as compared with the control (Figure 2, A–C). HnRNPA1 knockdown in the *KRAS^G12D^* PDAC cells was followed by decreased hnRNPA1 expression levels in the corresponding EVs and hnRNPA1 overexpression induced hnRNPA1 enrichment in the *KRAS^G12D^* PDAC cell–secreted EVs, while the expression levels of hnRNPA1 in EVs changed slightly after altering the cellular hnRNPA1 expression in PDAC cells with other *KRAS* subtypes (Figure 2, D and E, and Supplemental Figure 4, E–L). The EVs secreted by hnRNPA1-overexpressing *KRAS^G12D^* PDAC cells significantly enhanced HLEC tube formation and migration as compared with the control group, whereas hnRNPA1-overexpressing *KRAS^WT^* PDAC cell–secreted EVs exhibited slight effects on the tube formation and migration of HLECs (Figure 2F and Supplemental Figure 4, M and N). Conversely, hnRNPA1 downregulation in the EVs secreted by *KRAS^G12D^* PDAC cells abolished their abilities to induce HLEC tube formation and migration (Figure 2G and Supplemental Figure 4, O and P). These results demonstrate that EV-packaged hnRNPA1 secreted by *KRAS^G12D^* PDAC cells facilitates the tube formation and migration of HLECs to induce lymphangiogenesis in vitro.

### EV-packaged hnRNPA1 induces LN metastasis of KRAS^G12D^ PDAC in vivo.

To explore whether hnRNPA1 was involved in *KRAS^G12D^*-induced LN metastasis of PDAC in vivo, we established the popliteal lymphatic metastasis model through implanting hnRNPA1-overexpressing or -knockdown PANC-1 cells (*KRAS^G12D^*) or BxPC-3 cells (*KRAS^WT^*) and corresponding control cells separately. HnRNPA1 overexpression significantly promoted PANC-1 cell metastasis to the popliteal LNs and hnRNPA1 knockdown suppressed the LN metastasis of PANC-1 cells, as indicated by an in vivo imaging system (IVIS), while the alteration of hnRNRA1 expression in BxPC-3 produced only rare effects on LN metastasis (Supplemental Figure 5A). Larger LNs were detected in the hnRNPA1-overexpressing PANC-1 group as compared with the control PANC-1 group, whereas decreased LN volumes were detected in the hnRNPA1-knockdown group (Supplemental Figure 5B). Moreover, the microlymphatic vessel density in primary tumors was dramatically increased by hnRNPA1 overexpression and reduced by hnRNPA1 knockdown, while either hnRNPA1 overexpression or knockdown in BxPC-3 only slightly affected the quantification of microlymphatic vessels (Supplemental Figure 5C), indicating that hnRNPA1 is involved in *KRAS^G12D^*-induced LN metastasis of PDAC.

**Figure 3:**
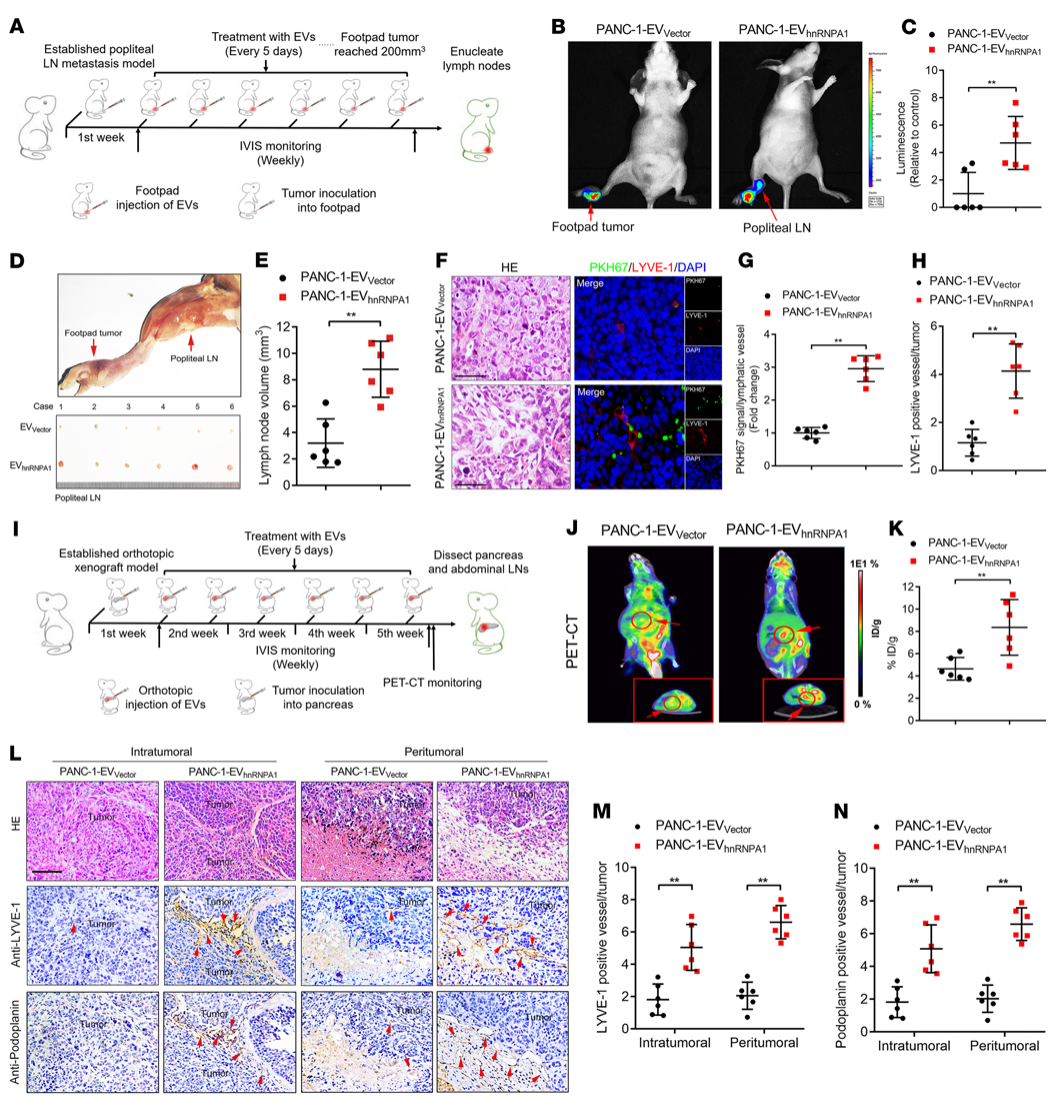
EV-packaged hnRNPA1 induces LN metastasis of *KRAS^G12D^* PDAC in vivo. (**A**) Schematic representation of the establishment of the popliteal lymphatic metastasis model. (**B** and **C**) Representative images (**B**) and quantification (**C**) of bioluminescence of the popliteal metastatic LNs (*n =* 6 per group). Red arrows: Footpad tumor and metastatic popliteal LNs. The 2-tailed Student’s *t* test was used. (**D** and **E**) Representative image (**D**) of popliteal lymphatic metastasis model. Quantification (**E**) of the popliteal LN volume is shown. Red arrows: Footpad tumor and metastatic popliteal LNs. The 2-tailed Student’s *t* test was used. (**F**–**H**) Representative H&E-stained and immunofluorescence images (**F**) and quantification of PKH67-labeled EVs (**G**) or LYVE-1–positive lymphatic vessel density (**H**) in footpad tumors. Scale bars: 50 μm. The 2-tailed Student’s *t* test was used. (**I**) Schematic representation of orthotopic xenograft model establishment. (**J** and **K**) Representative images of PET-CT images of orthotopic tumors. Red arrows: Orthotopic tumor. ^18^FDG accumulation in the pancreas was assessed (*n =* 6 per group). ID, injected dose. The 2-tailed Student’s *t* test was used. (**L**–**N**) Representative H&E-stained and IHC images (**L**) and quantification (**M** and **N**) of LYVE-1–positive or podoplanin-positive lymphatic vessel density in orthotopic tumors (*n =* 6 per group). Scale bar: 50 μm. The 2-tailed Student’s *t* test was used. Data are presented as mean ± SD; 3 independent experiments were performed. *P < 0.05, **P < 0.01.

As we indicated that hnRNPA1 fostered the lymphangiogenesis of *KRAS^G12D^* PDAC through the EV-packaged form, we further evaluated the effect of EV-packaged hnRNPA1 on LN metastasis of *KRAS^G12D^* PDAC in an EV-induced popliteal lymphatic metastasis model (Figure 3A). Subsequently, the mice were intratumorally treated with PBS, EVs secreted by PDAC cell lines with different *KRAS* subtypes (*KRAS^WT^*: BxPC-3-EV_Vector_; *KRAS^G12V^*: Capan-2-EV_Vector_; *KRAS^G12D^*: PANC-1-EV_Vector_), or EVs secreted by hnRNPA1-overexpressing PANC-1 cells (PANC-1-EV_hnRNPA1_) (Supplemental Figure 5, D–F). IVIS showed that PANC-1-EV_hnRNPA1_ significantly promoted PANC-1 cell metastasis to the popliteal LNs when compared with the PANC-1-EV_Vector_, while treatment with PBS or EVs secreted by PDAC cell lines with other *KRAS* subtypes (BxPC3-EV_Vector_ or Capan-2-EV_Vector_) had only rare effects on the popliteal LN metastasis of mice (Figure 3, B and C, and Supplemental Figure 5, G–I). Moreover, the PANC-1-EV_hnRNPA1_ group had significantly increased the popliteal LN volumes, while PANC‑1‑EV_Vector_ slightly enlarged the popliteal LNs when compared with the PBS, BxPC3-EV_Vector_, and Capan-2-EV_Vector_ groups (Figure 3, D and E, and Supplemental Figure 5J). Increased LN metastatic rates were observed in mice treated with PANC-1-EV_hnRNPA1_ as compared with those that received PANC-1-EV_Vector_ treatment (Supplemental Figure 5K). Importantly, confocal microscopy revealed significant internalization of PKH67-labeled EVs by lymphatic vessels in the PANC-1-EV_hnRNPA1_ group, which increased the number of microlymphatic vessels as indicated by representative markers of lymphangiogenesis, including LYVE-1, podoplanin, VEGFR3, CD31, and NRP2 in the intratumoral and peritumoral regions of the primary tumors. Since infiltrated cells in the TME have been previously reported to contribute to the lymphangiogenesis and promote LN metastasis (22, 23), we evaluated whether the abundant cells in the TME of PDAC, including cancer-associated fibroblasts (CAFs) and tumor-associated macrophages (TAMs), were required for EV-packaged-hnRNPA1–mediated lymphangiogenesis. The results showed that PANC-1-EV_hnRNPA1_ rarely affected the infiltration of α-smooth muscle actin–positive (α-SMA–positive) CAFs and CD68-positive TAMs in the primary tumor as compared with the control (Figure 3, F–H, and Supplemental Figure 5, L–P), suggesting that EV-packaged hnRNPA1 directly triggered lymphangiogenesis of *KRAS^G12D^* PDAC independent of the infiltrated cells, including CAFs and TAMs in the TME. Together, our results demonstrate that EV-packaged hnRNPA1 induces *KRAS^G12D^* PDAC lymphangiogenesis and LN metastasis.

To simulate the anatomy and physiology of LN metastasis in vivo, we established an orthotopic xenograft model to investigate the role of EV-packaged hnRNPA1 in LN metastasis of *KRAS^G12D^* PDAC (Figure 3I). Positron emission tomography–computed tomography (PET-CT) scanning showed that the PANC-1-EV_hnRNPA1_ group had higher accumulation of ^18^F-fluorodeoxyglucose (^18^FDG) than the PANC-1-EV_Vector_ group (Figure 3, J and K, and Supplemental Figure 6A), suggesting that EV-packaged hnRNPA1 promoted the orthotopic tumorigenicity of *KRAS^G12D^* PDAC cells. Given that the peripancreatic LNs in the abdomen, including the pyloric, hilar, and superior mesenteric LNs, represent the most common drainage LNs of PDAC in mice (24), we enucleated them to evaluate the effects of EV-packaged hnRNPA1 on LN metastasis of *KRAS^G12D^* PDAC. The overexpression of EV-packaged hnRNPA1 significantly facilitated PANC-1 cell metastasis to the peripancreatic LNs (Supplemental Figure 6, B–E, and Supplemental Table 2). Furthermore, PANC-1-EV_hnRNPA1_ treatment promoted lymphangiogenesis in the primary tumor and the subcapsular sinus of the peripancreatic LNs (Figure 3, L–N, and Supplemental Figure 6, F and G). Additionally, only rare differences in metastasis to the liver or omentum was found between the PANC-1-EV_Vector_ and PANC-1-EV_hnRNPA1_ groups (Supplemental Figure 6, H and I), suggesting the specific role of EV-packaged hnRNPA1 in LN metastasis rather than distant metastasis. Collectively, these findings demonstrate that EV-packaged hnRNPA1 promotes *KRAS^G12D^* PDAC lymphangiogenesis and LN metastasis in vivo.

### KRAS signaling–induced SAE1 overexpression catalyzes hnRNPA1 SUMOylation.

**Figure 4:**
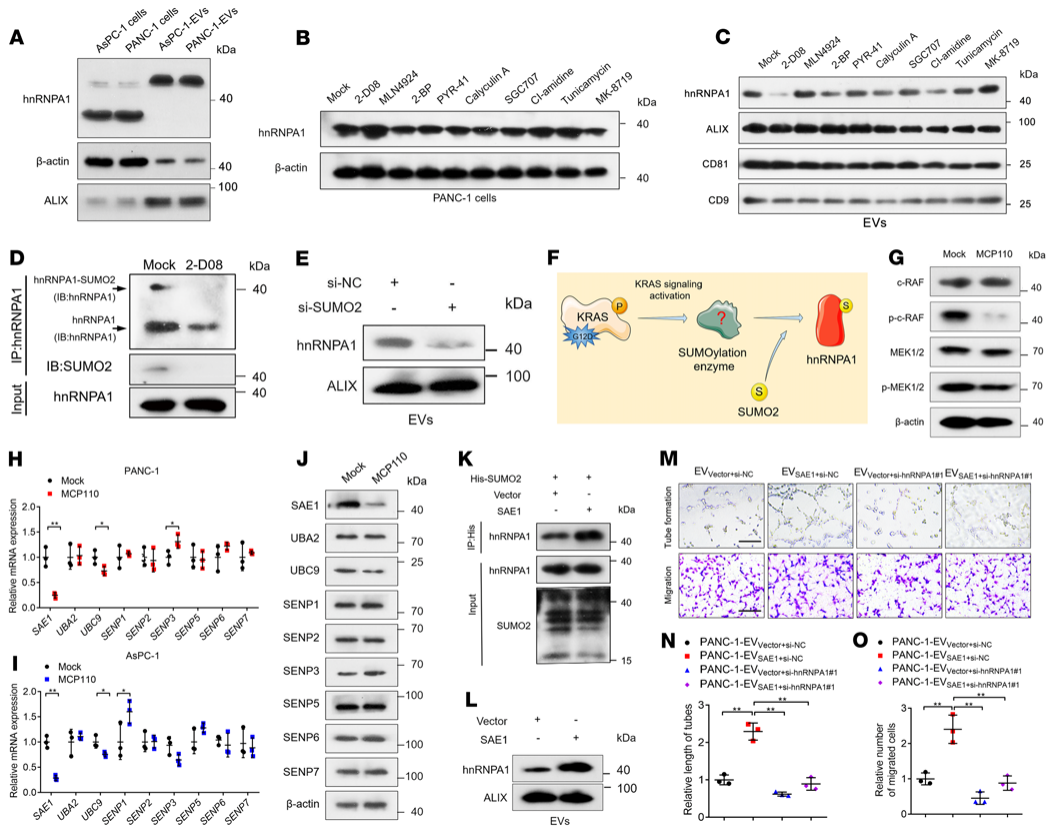
KRAS signaling–induced *SAE1* overexpression catalyzes the SUMOylation of hnRNPA1. (**A**) Western blotting analysis of hnRNPA1 expression in PDAC cells and the corresponding EVs. (**B** and **C**) Western blotting assessment of hnRNPA1 expression in PANC-1 cells (**B**) and the corresponding EVs (**C**) after treatment with PBS or indicated inhibitors of PTMs. (**D**) IP assessment of SUMO2 binding to hnRNPA1 after 2-D08 treatment. IB, immunoblot. (**E**) Western blotting analysis of hnRNPA1 expression in EVs secreted by PANC-1 cells after *SUMO2* silencing. (**F**) Schematic illustration of the hypothesis of *KRAS^G12D^*-induced SUMOylation of hnRNPA1. (**G**) Western blotting analysis of the KRAS downstream pathway in PANC-1 cells after treatment with MCP110. (**H**–**J**) qRT-PCR (**H** and **I**) and Western blotting analysis (**J**) of SUMOylation enzyme expression in PDAC cells after MCP110 treatment. The 2-tailed Student’s *t* test was used. (**K**) Co-IP assessment of SUMO2 binding to hnRNPA1 after SAE1 overexpression. (**L**) Western blotting analysis of hnRNPA1 expression in PANC-1 cell–secreted EVs after SAE1 overexpression. (**M**–**O**) Representative images (**M**) and quantification of tube formation (**N**) and migration (**O**) of HLECs treated with indicated EVs. Scale bars: 100 μm. One-way ANOVA followed by Dunnett’s test was used. Data are presented as mean ± SD of 3 independent experiments. *P < 0.05, **P < 0.01.

As we indicated that EV-packaged hnRNPA1 overexpression induced lymphangiogenesis and LN metastasis of *KRAS^G12D^* PDAC, we explored the molecular mechanism triggering hnRNPA1 enrichment in *KRAS^G12D^* PDAC cell–secreted EVs. Interestingly, we found that EV-packaged hnRNPA1 had a higher molecular weight (>40 kDa) when compared with the hnRNPA1 in the cells (<40 kDa) (Figure 4A), suggesting that hnRNPA1 in *KRAS^G12D^* PDAC cell–secreted EVs underwent posttranslational modification (PTM). Then, we used inhibitors targeting various PTMs to detect the vital PTM involved in the high hnRNPA1 enrichment in *KRAS^G12D^* PDAC cell–secreted EVs. Only 2-D08, a specific inhibitor of SUMOylation, significantly decreased hnRNPA1 expression levels in the PDAC cell–secreted EVs, while hnRNPA1 expression in the PDAC cells was only slightly increased (Figure 4, B and C). Mass spectrometry (MS) analysis of the hnRNPA1 coimmunoprecipitation (co-IP) products showed that 2-D08 significantly suppressed the attachment of SUMO2, a SUMOylation modifier, to hnRNPA1 (Supplemental Figure 7, A and B), which was validated by Western blotting analysis (Figure 4D). Moreover, SUMO2 knockdown greatly downregulated hnRNPA1 expression levels in the PDAC cell–secreted EVs (Figure 4E). These results suggest that SUMO2 modification of hnRNPA1 is essential for hnRNPA1 loading into EVs.

Next, we investigated the mechanism triggering hnRNPA1 SUMOylation in *KRAS^G12D^* PDAC cells. Accumulating evidence has demonstrated that the *KRAS^G12D^* mutation predominantly causes the rapidly accelerated fibrosarcoma/mitogen–activated protein kinase/extracellular regulated protein kinase (RAF/MEK/ERK) signaling pathway to promote PDAC progression (25, 26). Accordingly, we used a small-molecule inhibitor targeting the KRAS/RAF signaling pathway, MCP110, to evaluate whether *KRAS^G12D^*-induced RAF signaling activation stimulates hnRNPA1 SUMOylation in *KRAS^G12D^* PDAC cells (Figure 4F). MCP110 significantly reduced RAF and MEK1/2 phosphorylation without affecting the total levels of RAF and MEK1/2 (Figure 4G), suggesting the successful inhibition of the KRAS/RAF signaling pathway. Among the multiple SUMOylation-related enzymes, the expression of SAE1, the crucial E1 SUMO-activating enzyme for SUMOylation modification (27), was significantly decreased after MCP110 treatment in the *KRAS^G12D^* PDAC cells (Figure 4, H–J). Moreover, overexpressing SAE1 significantly promoted SUMO2 modification of hnRNPA1 and facilitated hnRNPA1 packaging into the EVs (Figure 4, K and L). The in vitro experiments showed that SAE1 overexpression enhanced the abilities of PDAC-secreted EVs to induce HLEC tube formation and migration, which was reversed by downregulating hnRNPA1 expression in the PDAC-secreted EVs (Figure 4, M–O). Collectively, these findings demonstrate that the *KARS^G12D^* mutation upregulated SAE1 expression to induce the SUMOylation and EV sorting of hnRNPA1.

### HnRNPA1 is SUMOylated at the lysine 113 residue by SAE1.

**Figure 5:**
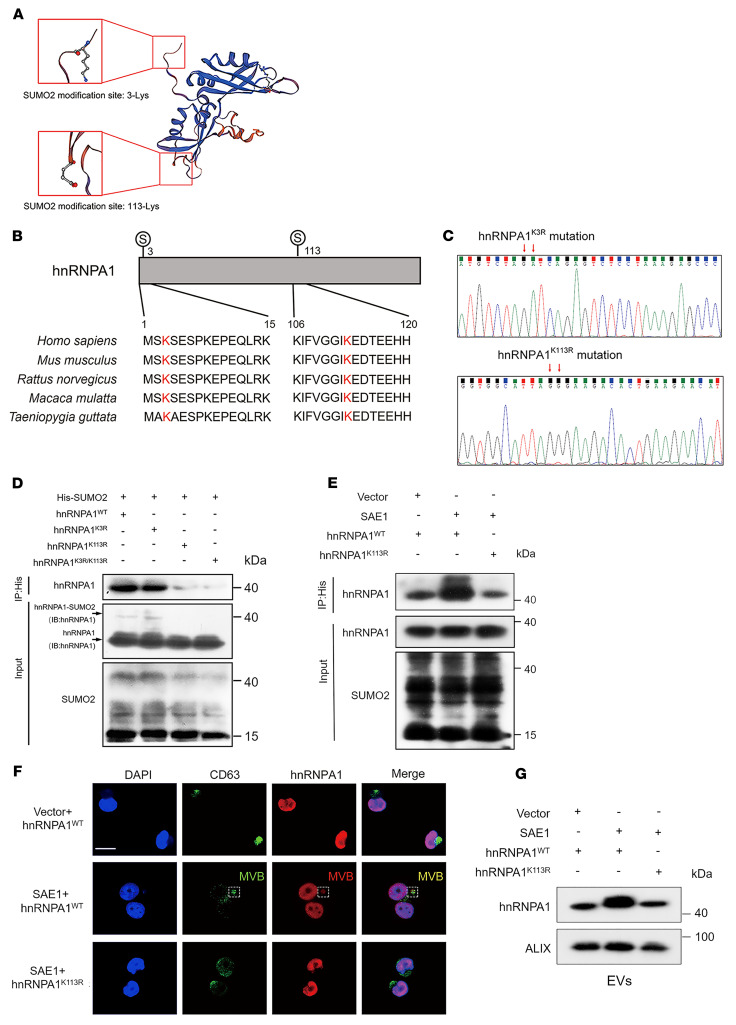
HnRNPA1 is SUMOylated at residue K113. (**A**) Schematic illustration of the predicted SUMO2 binding sites on hnRNPA1 obtained from GST-SUMO. (**B**) Sequence alignment of hnRNPA1 homologs in various species. (**C**) Sequencing evaluation of the hnRNPA1^K3R^ and hnRNPA1^K113R^ mutations. (**D** and **E**) Co-IP assays assessing the SUMO2 binding sites on hnRNPA1 and its regulation by SAE1. IB, immunoblot. (**F**) Representative immunofluorescence images of hnRNPA1 accumulation in CD63-positive MVBs in PANC-1 cells. Scale bar: 5 μm. (**G**) Western blotting analysis of hnRNPA1 expression in indicated EVs.

Considering that the modification residues have been implicated in the effects of SUMOylation on its target proteins (28), we used GPS-SUMO to predict 2 potential hnRNPA1 SUMO2 conjugation residues: lysine 3 (K3) and lysine 113 (K113) (Figure 5, A and B), which were then substituted with arginine (R) (hnRNPA1^K3R^, hnRNPA1^K113R^, hnRNPA1^K3R/K113R^) (Figure 5C and Supplemental Figure 7, C and D). HnRNPA1^K113R^ inhibited hnRNPA1 SUMOylation (Figure 5D), indicating that hnRNPA1 was predominantly SUMOylated at K113. Overexpressing SAE1 increased hnRNPA1 K113 SUMOylation (Figure 5E). Moreover, upregulating SAE1 enhanced the accumulation of hnRNPA1 in CD63-positive multivesicular bodies (MVBs) and subsequently facilitated hnRNPA1 loading into EVs (Figure 5, F and G). The hnRNPA1^K113R^ mutation significantly suppressed hnRNPA1 enrichment in the MVBs and decreased hnRNPA1 enrichment in the EVs (Figure 5, F and G), confirming that SAE1-induced SUMO2 binding with hnRNPA1^K113^ was essential for hnRNPA1 packaging into EVs.

### SUMOylation of hnRNPA1 enables its packaging into EVs by interacting with TSG101.

**Figure 6:**
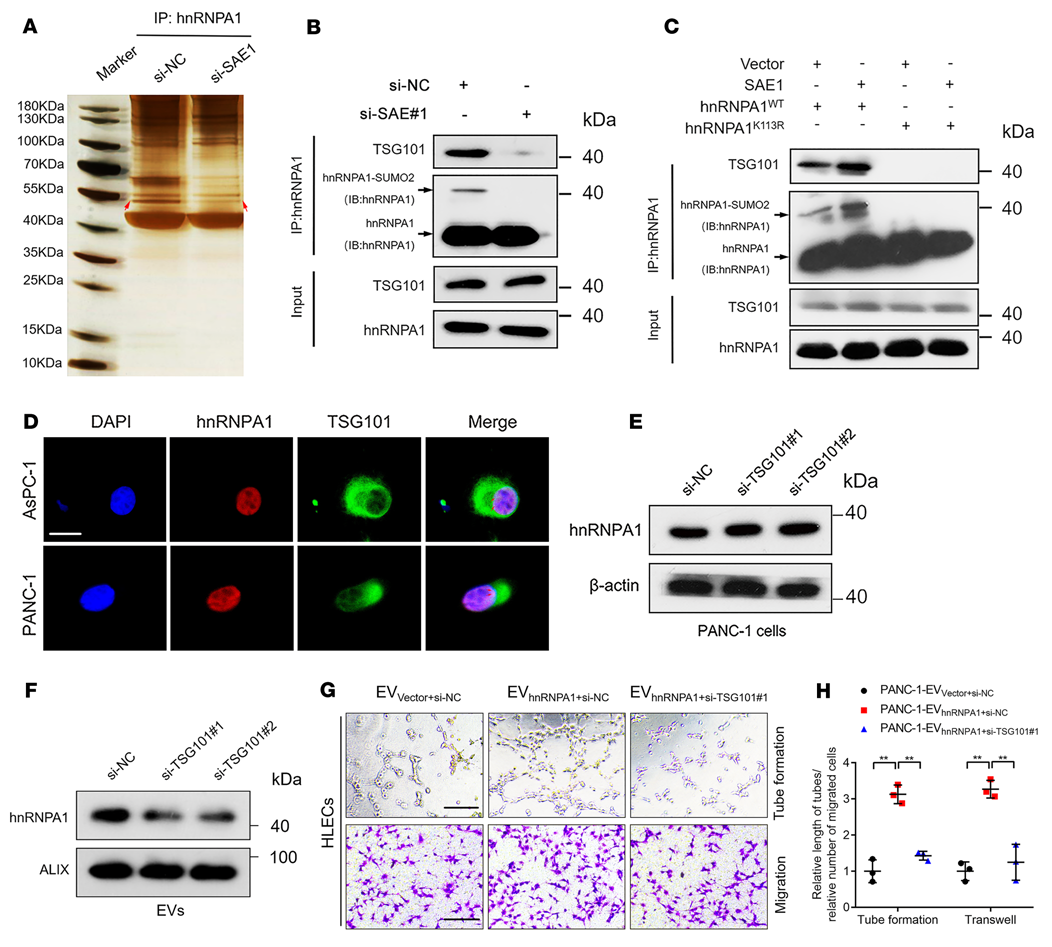
SUMOylated hnRNPA1 is packaged into EVs by interacting with TSG101. (**A** and **B**) Co-IP assay followed by silver staining (**A**) and Western blotting analysis (**B**) for detecting SUMOylated-hnRNPA1–interacting proteins in PANC-1 cells with or without SAE1 knockdown. IB, immunoblot. (**C**) Co‑IP assays analyzing the interaction of hnRNPA1 and TSG101 mediated by SAE1-induced SUMOylation on hnRNPA1. (**D**) Representative immunofluorescence images of hnRNPA1 and TSG101 colocalization in PDAC cells. Scale bar: 5 μm. (**E** and **F**) Western blotting analysis of hnRNPA1 expression in PANC-1 cells (**E**) and corresponding EVs (**F**) after TSG101 knockdown. (**G** and **H**) Representative images and quantification of tube formation and migration of HLECs treated with indicated EVs. Scale bars: 100 μm. One-way ANOVA followed by Dunnett’s test was used. Data are presented as mean ± SD of 3 independent experiments. **P < 0.01.

Since the interactions between proteins contribute to their subcellular location and extracellular exportation (29), we determined the binding partner of SUMOylated hnRNPA1. Co-IP assays followed by silver staining detected an obvious band of 44–55 kDa enriched by hnRNPA1 co-IP in PDAC cells treated with negative control siRNA compared with SAE1-depleted PDAC cells, which MS and Western blotting analyses identified as tumor susceptibility 101 (TSG101) (Figure 6, A and B, and Supplemental Figure 8, A and B). SAE1 overexpression promoted hnRNPA1’s interaction with TSG101, which was critically inhibited by the hnRNPA1^K113R^ mutation (Figure 6C), confirming that SAE1-induced SUMOylated hnRNPA1 bound directly with TSG101. Moreover, hnRNPA1 and TSG101 were colocalized in the nuclei of PDAC cells (Figure 6D). As TSG101 is a crucial component of the endosomal sorting complex responsible for transport (ESCRT) and triggers EV synthesis by loading proteins into EV precursors (30, 31), we evaluated whether it mediated hnRNPA1 packaging into EVs. TSG101 knockdown significantly decreased hnRNPA1 enrichment in PDAC cell–secreted EVs without affecting cellular hnRNPA1 expression, while hnRNPA1 was significantly upregulated in EVs secreted by TSG101-overepressing cells (Figure 6, E and F, and Supplemental Figure 8, C and D), suggesting that TSG101 promoted hnRNPA1 packaging into EVs. Furthermore, we assessed whether TSG101 was essential for EV transmission of hnRNPA1 for inducing lymphangiogenesis in PDAC. The results showed that TSG101 knockdown greatly inhibited EV-packaged-hnRNPA1–induced HLEC tube formation and migration (Figure 6, G and H). Altogether, these findings demonstrate that SUMOylation on hnRNPA1^K113^ triggers its packaging into EVs with the assistance of TSG101 in *KRAS^G12D^* PDAC.

### EV-packaged hnRNPA1 is delivered to HLECs to induce lymphangiogenesis.

**Figure 7:**
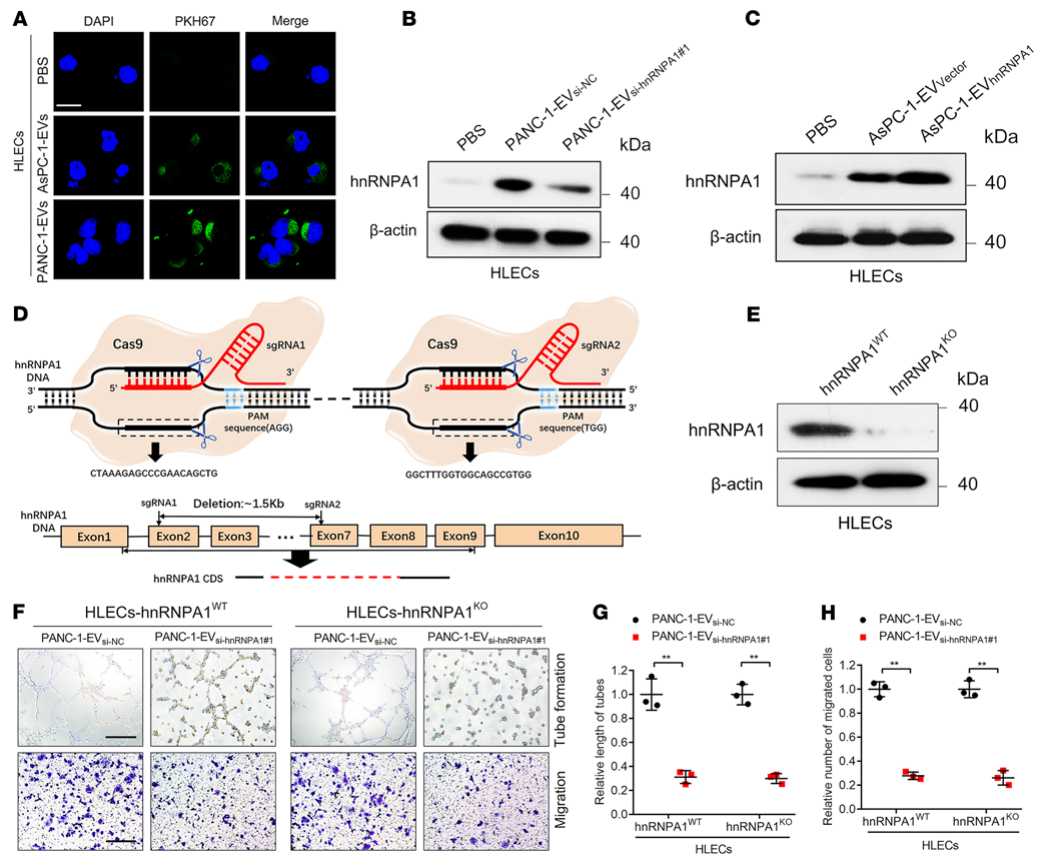
EV-packaged hnRNPA1 is delivered to HLECs. (**A**) Representative fluorescence images of HLECs after incubation with PKH67-labeled EVs. Scale bar: 5 μm. (**B** and **C**) Western blotting analysis of hnRNPA1 expression in PBS- or EV-treated HLECs. (**D**) Schematic representation of CRISPR/Cas9-mediated hnRNPA1 deletion in HLECs. (**E**) Western blotting analysis validation of hnRNPA1 knockout in HLECs. (**F**–**H**) Representative images (**F**) and quantification of tube formation (**G**) and migration (**H**) of EV-treated hnRNPA1^WT^ or hnRNPA1^KO^ HLECs. Scale bars: 100 μm. The 2-tailed Student’s *t* test was used. Data are presented as mean ± SD of 3 independent experiments. **P < 0.01.

Since our results indicated that SUMOylated hnRNPA1 was packaged into EVs via interaction with TSG101 and subsequently promotes *KRAS^G12D^* PDAC lymphangiogenesis, we investigated how EV-packaged hnRNPA1 regulated HLECs. PDAC cell–secreted EVs were labeled with PKH67 and incubated with HLECs. Confocal microscopy revealed that the green fluorescence signal from the PKH67-labeled EVs was present in the HLEC cytoplasm, while no such signal was detected in the control group (Figure 7A). Moreover, HLECs treated with PANC-1-EV_si-hnRNPA1#1_ (PANC-1 cell EVs with hnRNPA1 silencing) exhibited lower hnRNPA1 expression levels than the control group, while hnRNPA1 overexpression was detected in HLECs treated with PANC-1‑EV_hnRNPA1_ (Figure 7, B and C), indicating that EV-packaged hnRNPA1 had been delivered to the HLECs.

To exclude the possibility that *KRAS^G12D^* PDAC cell–secreted EVs promoted HLEC tube formation and migration by inducing endogenous hnRNPA1 transcription in HLECs, we utilized the clustered regularly interspaced short palindromic repeats/CRISPR-associated protein 9 (CRISPR/Cas9) approach to construct an endogenous hnRNPA1-knockout (hnRNPA1^KO^) HLEC line (Figure 7, D and E). EV-packaged-hnRNPA1 knockdown suppressed the tube formation and migration of hnRNPA1^KO^ HLECs induced by PDAC cell–secreted EVs, while EV-packaged-hnRNPA1 overexpression significantly promoted hnRNPA1^KO^ HLEC tube formation and migration (Figure 7, F–H, and Supplemental Figure 8, E–J). These results are consistent with those obtained in wild-type hnRNPA1 (hnRNPA1^WT^) HLECs in vitro, suggesting that PDAC-secreted EVs regulated HLEC function by transmitting EV-packaged hnRNPA1 rather than by activating hnRNPA1 transcription. Taken together, our findings demonstrate that *KRAS^G12D^* PDAC cell–secreted EVs induce lymphangiogenesis by delivering EV-packaged hnRNPA1 to HLECs.

### SUMOylation of EV-packaged hnRNPA1 enhances prospero homeobox 1 mRNA stability in HLECs.

It has been proposed that VEGF-C represents the core regulator for inducing tumor lymphangiogenesis (32). Accordingly, we analyzed whether hnRNPA1 participates in regulating VEGF-C to promote the lymphangiogenesis of PDAC. The results showed that either overexpression or knockdown of hnRNPA1 affected the VEGF-C expression and secretion of PDAC cells (Supplemental Figure 9, A–D). Since VEGFR3 in HLECs has been well characterized as the receptor for VEGF-C to induce the sprouting of lymphatic vessels (33), we further constructed CRISPR/Cas9-mediated VEGFR3-knockout HLECs to analyze whether EV-packaged hnRNPA1 triggered lymphangiogenesis independent of VEGF-C signaling (Supplemental Figure 9E). The tube formation and migration of HLECs were significantly inhibited after VEGFR3 knockout, while EV-packaged-hnRNPA1 overexpression still promoted the tube formation and migration of VEGFR3-knockout HLECs (Supplemental Figure 9, F–H), suggesting that hnRNPA1 promotes lymphangiogenesis and LN metastasis independent of VEGF-C.

**Figure 8:**
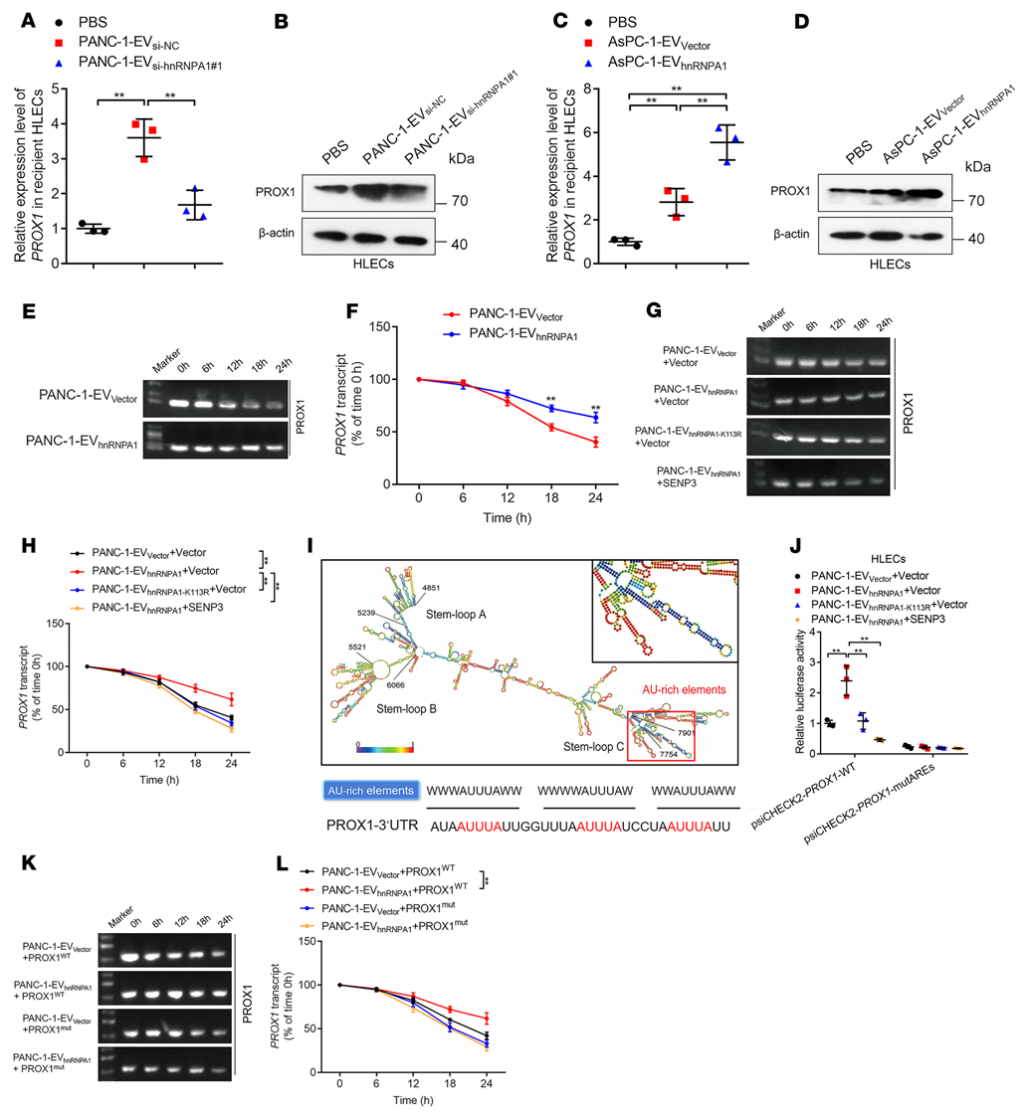
EV-packaged hnRNPA1 enhances *PROX1* mRNA stability in HLECs. (**A**–**D**) qRT-PCR (**A** and **C**) and Western blotting analysis (**B** and **D**) of PROX1 expression in PBS- or EV-treated HLECs. One-way ANOVA followed by Dunnett’s test was used. (**E**–**H**) Representative agarose electrophoresis images and quantification of actinomycin assays for *PROX1* mRNA in indicated EV-treated HLECs with or without *SENP3* overexpression. The 2-tailed Student’s *t* test (**F**) or 1-way ANOVA followed by Dunnett’s test was used (**H**). (**I**) Schematic illustration of the AREs in the *PROX1* mRNA 3′-UTR. (**J**) Dual-luciferase assays of wild-type or ARE-mutated *PROX1* in HLECs. One-way ANOVA followed by Dunnett’s test was used. (**K** and **L**) Representative agarose electrophoresis images (**K**) and quantification (**L**) of actinomycin assays for *PROX1* mRNA in EV-treated HLECs with or without ARE mutation in the *PROX1* mRNA. One-way ANOVA followed by Dunnett’s test was used. Data are presented as mean ± SD of 3 independent experiments. **P < 0.01.

Prospero homeobox 1 (PROX1) is considered a key player in lymphatic endothelium maintenance and facilitates lymphatic vessel development during lymphangiogenesis (5, 6). Therefore, we investigated PROX1 expression in EV-packaged-hnRNPA1–treated HLECs. The results showed that PROX1 expression correlated positively with hnRNPA1 expression levels in the *KRAS^G12D^* PDAC cell–secreted EVs, while EVs secreted by hnRNPA1-overexpressing PDAC cells with other *KRAS* subtypes or the stromal cells only rarely affected PROX1 expression in HLECs (Figure 8, A–D, and Supplemental Figure 10A), suggesting that PROX1 was the downstream target of EV-packaged hnRNPA1 secreted by *KRAS^G12D^* PDAC cells. Dual-luciferase assays for determining the molecular mechanism of EV-packaged hnRNPA1 in regulating PROX1 expression showed that EV-packaged hnRNPA1 had little effect on the *PROX1* promoter region, while a significant increase in luciferase activity was observed when activating the *PROX1* 3′-untranslated region (3′-UTR) (Supplemental Figure 10, B–E). Actinomycin assays also revealed a positive correlation between EV-packaged hnRNPA1 expression levels and the half-life of *PROX1* mRNA (Figure 8, E and F, and Supplemental Figure 10F), suggesting that EV-packaged hnRNPA1 upregulated PROX1 expression by stabilizing *PROX1* mRNA rather than by affecting *PROX1* transcription activity. As *KRAS^G12D^* PDAC cell–secreted EV-packaged hnRNPA1 was predominantly SUMOylated, we used SUMO-specific peptidase 3 (SENP3) to inhibit hnRNPA1 SUMOylation in *KRAS^G12D^* PDAC cells, which significantly attenuated the ability of EV-packaged hnRNPA1 to stabilize *PROX1* mRNA (Figure 8, G and H, and Supplemental Figure 10G). Moreover, the hnRNPA1^K113R^ mutation significantly impaired EV-packaged-hnRNPA1–induced stabilization of *PROX1* mRNA (Figure 8, G and H, and Supplemental Figure 10G), validating that the SUMOylation of EV-packaged hnRNPA1 promoted its effect on *PROX1* mRNA stability. Given that the adenylate- and uridylate-rich (AU-rich) elements (AREs) in the mRNA 3′-UTR are common determinants of RNA stability in mammalian cells (34), we analyzed whether EV-packaged hnRNPA1 regulated *PROX1* mRNA stability via interaction with *PROX1* AREs. RNA IP (RIP) showed that EV-packaged hnRNPA1 bound directly to *PROX1* mRNA, which was abolished by inhibiting hnRNPA1 SUMOylation (Supplemental Figure 10, H and I). AREsite2 analysis led to the identification of an AU-rich region that contains 3 AUUUA core pentamers in the *PROX1* 3′-UTR (Figure 8I). Dual-luciferase reporter assays revealed that EV-packaged hnRNPA1 increased *PROX1* promoter luciferase activity via SUMOylation, while inducing mutation in the *PROX1* AREs abolished the effects of EV-packaged hnRNPA1 on the *PROX1* promoter luciferase activity (Figure 8J and Supplemental Figure 10J), suggesting that EV-packaged hnRNPA1 interacted directly with the *PROX1* AREs. Moreover, the actinomycin assays demonstrated that ARE mutations inhibited the effect of EV-packaged hnRNPA1 on *PROX1* mRNA stability (Figure 8, K and L, and Supplemental Figure 10K).

### EV-packaged hnRNPA1 promotes PROX1-induced lymphangiogenesis and LN metastasis.

**Figure 9:**
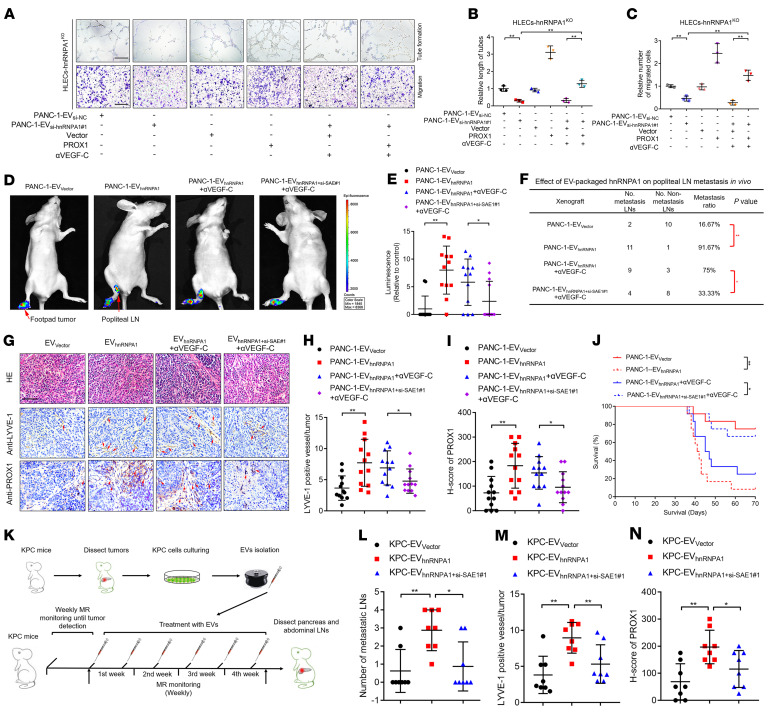
PROX1 is indispensable for EV-packaged-hnRNPA1–induced lymphangiogenesis and LN metastasis of *KRAS^G12D^* PDAC. (**A–C**) Representative images and quantification of tube formation and migration of PANC-1-EV_si-NC_– or PANC-1-EV_si-hnRNPA1#1_–treated hnRNPA1^KO^ HLECs with or without PROX1 overexpression and VEGF-C–neutralizing antibody. Scale bars: 100 μm. One-way ANOVA followed by Dunnett’s test was used. (**D** and **E**) Representative images and quantification of bioluminescence of the popliteal metastatic LNs (*n =* 12 per group). One-way ANOVA followed by Dunnett’s test was used. (**F**) The analysis of LN metastasis rate in indicated groups of popliteal LN metastasis model. The χ^2^ test was used. (**G**–**I**) Representative H&E-stained and IHC images and quantification of LYVE-1–positive lymphatic vessels and PROX1 expression in footpad tumors. Scale bar: 50 μm. One-way ANOVA followed by Dunnett’s test was used. (**J**) Kaplan-Meier curves for the nude mice. (**K**) Schematic representation of KPC mouse model establishment (*n =* 8 per group). One-way ANOVA followed by Dunnett’s test was used. (**L**) Quantification of the metastatic number of peripancreatic LNs. One-way ANOVA followed by Dunnett’s test was used. (**M** and **N**) Quantification of IHC analysis for LYVE-1–positive lymphatic vessels and PROX1 expression in pancreatic tumors. One-way ANOVA followed by Dunnett’s test was used. Data are presented as mean ± SD of 3 independent experiments. *P < 0.05, **P < 0.01.

As we determined that EV-packaged hnRNPA1 targeted HLECs to enhance *PROX1* mRNA stability, we investigated whether PROX1 was required for EV-packaged-hnRNPA1–induced lymphangiogenesis and LN metastasis. The in vitro assays revealed that reducing EV-packaged-hnRNPA1 expression levels abolished HLEC tube formation and migration induced by *KRAS^G12D^* PDAC cell–secreted EVs, while PROX1 overexpression reversed this effect even after VEGF-C had been blocked with VEGF-C–neutralizing antibody (αVEGF-C) (Figure 9, A–C). Conversely, PROX1 knockdown reversed EV-packaged-hnRNPA1–induced lymphangiogenesis in a VEGF-C–independent manner, indicating that EV-packaged hnRNPA1 facilitated lymphangiogenesis by upregulating PROX1 in HLECs independent of VEGF-C (Supplemental Figure 11, A–C).

Given that SUMOylation-driven EV transmission of hnRNPA1 was conducive to PDAC-secreted-EV–mediated PROX1 overexpression for triggering lymphangiogenesis, we explored whether it contributed to *KRAS^G12D^* PDAC LN metastasis. In vitro experiments revealed that ectopic hnRNPA1 expression in HLECs only slightly promoted the tube formation and migration of HLECs, while upregulating SAE1 to induce the SUMOylation of hnRNPA1 significantly triggered HLEC tube formation and migration. The hnRNPA1^K113R^ mutation significantly impaired the hnRNPA1-induced tube formation and migration of HLECs with or without SAE1 overexpression (Supplemental Figure 11, D–F). Moreover, a popliteal LN metastasis mouse model was constructed to show that EV-packaged-hnRNPA1 overexpression enhanced LN metastasis induced by PDAC-cell-secreted EVs. Downregulating SAE1 to suppress EV-packaged-hnRNPA1 transmission reversed these effects after αVEGF-C treatment in both groups (Figure 9, D and E). Compared with the PANC-1-EV_hnRNPA1_ plus αVEGF-C group, the PANC-1-EV_hnRNPA1+si-SAE1#1_ plus αVEGF-C group had reduced incidence of LN metastasis (Figure 9F). Blocking SUMOylation on hnRNPA1 through SAE1 knockdown also inhibited the EV-packaged-hnRNPA1–induced increase in LYVE-1-positive microlymphatic vessels and PROX1 expression in primary tumors in a VEGF-C–independent manner (Figure 9, G–I). Furthermore, mice in the PANC-1-EV_hnRNPA1+si-SAE1#1_ plus αVEGF-C group had prolonged survival time compared with those in the PANC-1-EV_hnRNPA1_ plus αVEGF-C group (Figure 9J).

*Kras^G12D/+^Trp53^R172H/+^Pdx-1*-Cre (KPC) mice are well characterized as a genetically engineered PDAC model system with autonomously growing tumors to mimic *KRAS^G12D^* mutation–induced PDAC progression (35). Therefore, we evaluated the effect of SUMOylation of EV-packaged hnRNPA1 on the regulation of PROX1 expression to induce LN metastasis of *KRAS^G12D^* PDAC in the KPC mouse model. The results showed that EVs overexpressing hnRNPA1 significantly promoted LN metastasis in KPC mice and the effect was reversed by inhibiting SAE1-induced SUMOylation, while only rare effects on liver or omentum metastasis were observed among these 3 groups (Figure 9, K and L, and Supplemental Figure 11, G–I). IHC analysis revealed that EV-packaged hnRNPA1 increased the LYVE-1–positive microlymphatic vessels and PROX1 expression in primary tumors, which was abolished by SAE1 knockdown (Figure 9, M and N). Taken together, these results indicate that EV-packaged hnRNPA1 promotes lymphangiogenesis and LN metastasis of *KRAS^G12D^* PDAC by upregulating PROX1 expression.

### The clinical relevance of EV-packaged hnRNPA1 in patients with LN metastatic PDAC.

**Figure 10:**
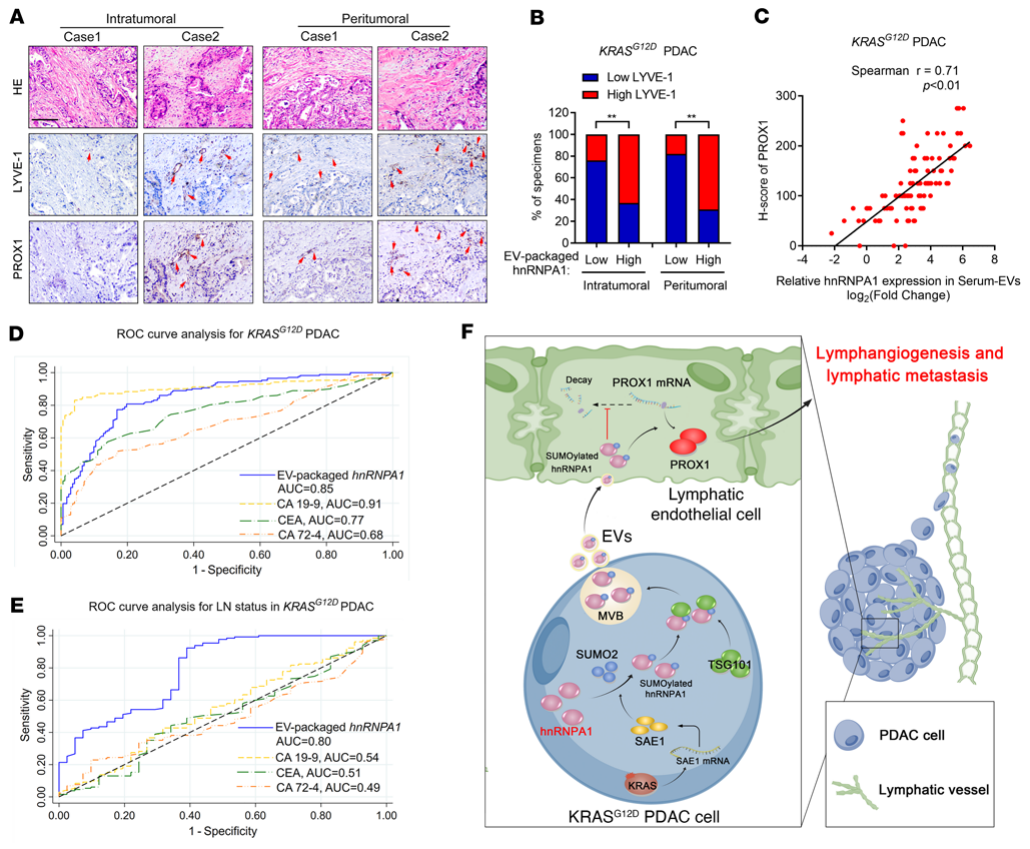
EV-packaged hnRNPA1 correlates with LN metastasis of patients with *KRAS^G12D^* PDAC. (**A**–**C**) Representative images (**A**) for determination of LYVE-1–positive lymphatic vessel density and PROX1 expression in *KRAS^G12D^* PDAC according to EV-packaged-hnRNPA1 expression. The percentages of IHC staining for LYVE-1–positive lymphatic vessel density and the correlation between EV-packaged hnRNPA1 and PROX1 expression were analyzed (**B** and **C**). Scale bar: 50 μm. The χ^2^ test was used. (**D** and **E**) ROC analysis of the diagnostic efficiency of serum EV-packaged hnRNPA1, CA19-9, CEA, and CA72-4 for *KRAS^G12D^* PDAC (**D**) or LN metastasis (**E**) of *KRAS^G12D^* PDAC. (**F**) Proposed model of KRAS signaling–induced SUMOylation of EV-packaged hnRNPA1 that mediates *PROX1* mRNA stability for facilitating *KRAS^G12D^* PDAC LN metastasis. Data are presented as mean ± SD of 3 independent experiments. **P < 0.01.

As EV-packaged molecules have been identified as potential biomarkers and therapeutic targets in various cancers (36), we evaluated the clinical relevance of EV-packaged hnRNPA1 in *KRAS^G12D^* PDAC at 2 independent clinical centers (96 patients from Sun Yat-Sen Memorial Hospital of Sun Yat-sen University, and 76 patients from Guangdong Provincial People’s Hospital). EVs were extracted from the serum samples of patients with *KRAS^G12D^* PDAC and healthy controls, which were identified by TEM and NTA analysis (Supplemental Figure 12, A and B). EV-packaged hnRNPA1 was overexpressed in serum EVs from the patients with *KRAS^G12D^* PDAC as compared with the healthy controls (Supplemental Figure 12, C–E). Kaplan-Meier survival analysis revealed that EV-packaged-hnRNPA1 expression levels correlated positively with poor prognosis in the patients (Supplemental Figure 12, F–K). Univariate and multivariate analyses identified EV-packaged hnRNPA1 as an independent prognostic factor of OS and DFS of PDAC patients (Supplemental Tables 3 and 4). Moreover, the patients with LN metastasis or advanced tumor stage had higher serum EV-packaged hnRNPA1, SAE1, and PROX1 expression levels (Supplemental Figure 12, L–P, and Supplemental Table 5). Patients with higher EV-packaged-hnRNPA1 expression levels had upregulated SAE1 and PROX1 expression that was accompanied by increased microlymphatic vessel numbers (Figure 10, A–C, and Supplemental Figure 12, Q and R). Importantly, receiver operating characteristic (ROC) analysis revealed that EV-packaged hnRNPA1 exhibited superior diagnostic performance for *KRAS^G12D^* PDAC when compared with carcinoembryonic antigen (CEA) and carbohydrate antigen 72-4 (CA72-4), as indicated by the area under the curve (Figure 10D and Supplemental Figure 12S). EV-packaged hnRNPA1 was more effective for distinguishing LN-positive from LN-negative *KRAS^G12D^* PDAC than CA19-9, CEA, and CA72-4 (Figure 10, E and F). Our findings suggest that EV-packaged hnRNPA1 is a potential biomarker and therapeutic target in LN metastasis of *KRAS^G12D^* PDAC.

## Discussion

*KRAS* mutations are identified in more than 90% of patients with PDAC and tend to be associated with advanced stage and reduced OS of PDAC (9). There is increased physical interaction between tumor cells and endothelial cells in *KRAS^G12D^* PDAC, which might affect lymphangiogenesis and LN metastasis (13). However, the mechanism by which the *KRAS^G12D^* mutation regulates LN metastasis of PDAC remains unclear. In the present study, we uncovered that hnRNPA1 was upregulated in *KRAS^G12D^* PDAC cell–secreted EVs and promoted EV-mediated lymphangiogenesis and LN metastasis in both in vitro experiments and in xenografted, genetically engineered KPC mouse models. Moreover, hnRNPA1 was bound to SUMO2 as a result of *KRAS^G12D^*-induced SAE1 overexpression, which enhanced its physical interaction with TSG101 and triggered EV transmission of hnRNPA1. Subsequently, EV-packaged SUMOylated hnRNPA1 upregulated PROX1 expression in HLECs by stabilizing *PROX1* mRNA to facilitate the lymphangiogenesis of *KRAS^G12D^* PDAC. Our study clarifies a mechanism underlying *KRAS* mutant–related lymphangiogenesis and LN metastasis in PDAC through the induction of SUMOylation-related EV transmission, providing a perspective on clinical interventions for LN metastasis of *KRAS^G12D^* PDAC.

Lymphangiogenesis is well characterized as an essential step in LN metastasis in various cancers (37). Clinical evidence has shown that a high density of lymphatic vessels in PDAC is associated with increased LN metastasis and decreased OS (38, 39). Currently, the universally acknowledged mechanism for lymphangiogenesis mainly focuses on the VEGF-C–mediated lymphatic pathways (3, 40). Nevertheless, VEGF-C–targeted therapy fails to achieve satisfactory efficacy in 30% of PDAC with LN metastasis, encouraging further elucidation of the mechanism of lymphangiogenesis independent of VEGF-C in PDAC (3). Herein, we showed that lymphangiogenesis and LN metastasis occurred more frequently in *KRAS^G12D^* PDAC. *KRAS^G12D^* PDAC cells directly targeted *PROX1* mRNA in HLECs by transmitting SUMOylated hnRNPA1 in a VEGF-C–independent manner, after which SUMOylated hnRNPA1 directly bound to the *PROX1* ARE region to enhance *PROX1* mRNA stability, thereby promoting PDAC lymphangiogenesis. These findings demonstrate the VEGF-C–independent mechanism underlying LN metastasis of *KRAS^G12D^* PDAC by which SUMOylated hnRNPA1 regulates *PROX1* mRNA stability via EV transmission to induce lymphangiogenesis. In addition, accumulating evidence revealed that engineered EVs represent a prospective approach with high histocompatibility and targeted capacity for cancer therapy (41). Since mRNA stability is an important posttranscriptional regulatory process that allows rapid adjustment of the *PROX1* mRNA copy number and is crucial for driving the response of LECs (42), our results provide evidence for the potential application of PROX1-targeted engineered EVs in the treatment of LN metastatic PDAC.

EVs acquire various biological functions by packaging specific molecules during their biogenesis (43). It has been proposed that molecule packaging requires recognition by the ESCRT (44). The ESCRT consists of ESCRT-0, -I, -II, -III, and Vps4 complexes, directing protein incorporation into the endocytic system and the subsequent membrane abscission away from the cytosol to produce EVs (44, 45). ESCRT component activation and dysregulation alter EV contents and behaviors (46). However, the core regulator of ESCRT and its role in EV-induced PDAC LN metastasis remain unexplored. Herein, we found that TSG101 was specifically recruited by the lymphangiogenesis-driven protein hnRNPA1 and subsequently guided its transmission via EVs. Blocking SUMOylation eliminated TSG101-mediated encapsulation of EV-packaged hnRNPA1 and significantly suppressed the lymphangiogenesis and LN metastasis of PDAC both in vitro and in vivo. Additionally, it has been reported that hnRNPA1 participates in the sorting of RNAs into EVs to affect the various biological features of cancer (47, 48). Nevertheless, we found that treating simply with ectopic hnRNPA1 after the induction of its SUMOylation was able to facilitate lymphangiogenesis, implying that TSG101-induced EV transmission of SUMOylated hnRNPA1 represents a distinct mechanism independent of the role of hnRNPA1 in mediating the biomolecule transmission by EVs. These findings support the crucial role of the TSG101-dependent EV sorting pathway in PDAC lymphangiogenesis, suggesting a potential strategy for blocking EV transmission to suppress LN metastasis of PDAC.

SUMOylation represents a common biological event in protein regulation that affects protein stability, subcellular localization, or interaction ability (28, 49). Previously, we reported that SUMOylation induced by UBC9, the E2 ligase of SUMOylation, contributed to tumor lymphangiogenesis (6). Here, we identified that, in *KRAS^G12D^* PDAC, activation of KRAS signaling predominantly induced the SUMOylation pathway by upregulating SAE1 rather than UBC9, suggesting that SAE1 exhibits a more prominent function in *KRAS* mutation–induced SUMOylation to facilitate PDAC progression. As the most abundant E1 SUMO–activating enzyme in cancer, SAE1 initiates SUMOylation modification by catalyzing the C-terminal adenylation of SUMOs (28, 49). Binding with SUMOs mediates protein or RNA extracellular delivery, which induces cells in the TME to form a supportive environment for tumor metastasis (27). However, the role of SAE1 in triggering SUMOylation-mediated regulation of the TME to facilitate PDAC progression is largely unexplored. In the present study, we reported that the SAE1 overexpression induced by the KRAS/RAF signaling pathway sustained the SUMOylation of hnRNPA1 and triggered its packaging into EVs. Subsequently, it triggered the delivery of the aforementioned EVs into the TME to remodel the lymphatic vasculature. Blocking SAE1 abolished EV transmission of hnRNPA1 and inhibited PDAC LN metastasis in KPC mouse models. The identification of the machinery underlying *KRAS* mutant–driven SAE1-induced SUMOylation and its role in regulating EV-packaged-hnRNPA1–mediated lymphangiogenesis suggests that SAE1-mediated hnRNPA1 SUMOylation might represent a promising target for therapeutic strategies for suppressing *KRAS*-related LN metastasis of PDAC.

Another important finding was the improvement of LN metastasis diagnosis with the application of EV-packaged hnRNPA1. Currently, the assessment of LN status of PDAC mainly relies on imaging-based approaches, which are inaccurate, especially for early lesions (50). Therefore, monitoring LN metastasis in PDAC remains greatly challenging. Recently, there has been increased research attention on EV-packaged molecules because of their clinical significance as a convenient and noninvasive indicator in cancer diagnosis and risk stratification (15, 51–53). Herein, we found that EV-packaged hnRNPA1 was upregulated in the serum EVs from patients with PDAC and correlated positively with LN metastasis. Moreover, EV-packaged-hnRNPA1 expression levels exhibited greater accuracy than CEA or CA72-4 for differentiating patients with *KRAS^G12D^* PDAC from healthy controls, and had similar accuracy to that of CA19-9. Moreover, the detection of EV-packaged hnRNPA1 effectively distinguished patients with *KRAS^G12D^* PDAC with LN metastasis from those without LN metastasis, highlighting that EV-packaged-hnRNPA1 expression levels might be a feasible biomarker for overcoming the challenge of diagnosing LN metastasis in PDAC.

In summary, our findings provide essential information on the mechanism underlying KRAS-related regulation of lymphangiogenesis through the transmission of EV-packaged hnRNPA1 in a SUMOylation-dependent manner. Moreover, we found a positive correlation between EV-packaged hnRNPA1 and LN metastasis in patients with *KRAS^G12D^* PDAC and demonstrate its potential application in the clinical assessment of LN metastasis. Finally, our study highlights the role of *KRAS*-mutant–driven SUMOylation in triggering the delivery of EV-packaged hnRNPA1 to facilitate lymphangiogenesis. These results suggest hnRNPA1 as a potential therapeutic target for LN metastasis in *KRAS^G12D^* PDAC.

## Methods

### Patient samples.

A total of 186 patients with PDAC who had undergone surgery at Sun Yat-Sen Memorial Hospital of Sun Yat-sen University and another 76 patients with *KRAS^G12D^* mutation who had undergone surgery at Guangdong Provincial People’s Hospital were included. All PDAC tissues, confirmed by 2 pathologists independently, and paired normal adjacent tissues were acquired and quickly frozen in liquid nitrogen for protein extraction, or formalin-fixed and paraffin-embedded for IHC analysis. Blood samples were obtained from the patients with *KRAS^G12D^* PDAC and 172 paired healthy participants at the 2 independent centers.

### Cell lines and cell culture.

Human PDAC cell lines (*KRAS^G12D^*: PANC-1, AsPC-1; *KRAS^G12V^*: Capan-2; *KRAS^G12C^*: Mia-PaCa2; *KRAS^WT^*: BxPC-3) were obtained from American Type Culture Collection (ATCC). HPDE cells were obtained from Binsui Biotechnology. The HLECs were obtained from ScienCell Research Laboratories. The PANC-1 (ATCC, CRL-1469MET; RRID: CVCL_A4BT) and Capan-2 cells (ATCC, HTB-80; RRID: CVCL_0026) were maintained in DMEM (Invitrogen) containing 10% FBS. The AsPC-1 (ATCC, CRL-1682; RRID: CVCL_0152), BxPC-3 (ATCC, CRL-1687; RRID: CVCL_0186), Mia-PaCa2 (ATCC, CRM-CRL-1420; RRID: CVCL_0428), and HPDE cells (ATCC, HTX1979C) were maintained in RPMI 1640 medium (Invitrogen) containing 10% FBS. The HLECs (ScienCell Research Laboratories, 2500) were maintained in endothelial cell medium (ScienCell Research Laboratories) supplemented with 5% FBS. All cells were cultured at 37°C in humidified air with 5% CO_2_.

### Popliteal lymphatic metastasis model.

Four-week-old nude mice were purchased and fed at the Sun Yat-sen University animal center. Luciferase-expressing PANC-1 cells (1 × 10^6^) were injected into the right footpads of the mice, followed by intratumoral injection of 10 μg EVs in 50 μL PBS every 5 days. Popliteal lymphatic metastasis was monitored every week. When the primary tumor size was 200 mm^3^, the footpad tumors and popliteal LNs were excised, followed by formalin fixation and paraffin embedding for qRT-PCR and IHC analysis. Details are provided in the Supplemental Methods.

### Orthotopic xenograft model.

For the orthotopic xenograft model, 4-week-old nude mice were anesthetized with pentobarbital and maintained in the right-side lying position. An incision was made in the left lateral abdomen, and the pancreas was exposed by removing the spleen. Subsequently, 1 × 10^6^ PANC-1 cells were injected into the pancreas, and the abdomen was sutured. After the orthotopic xenograft model had been constructed, the mice received orthotopic injection of 50 μg EVs in 50 μL PBS using a 27-gauge needle once every 5 days. PET-CT was conducted to detect the tumors in the mice 4 weeks later. The primary tumors and the peripancreatic LNs in the abdomen, including the pyloric, hilar, and superior mesenteric LNs were enucleated for further analysis. The status of LNs was assessed by H&E staining and IHC analysis with anti-luciferase antibody.

### Genetically engineered model.

LSL-*Kras^G12D/+^* LSL-*Trp53^R172H/+^* *Pdx-1*-Cre mice were purchased from Shanghai Model Organisms. Eight-week-old mice were monitored by weekly MRI scans. After the pancreatic tumors were detected, the mice received orthotopic injection of 50 μg EVs in 50 μL PBS using a 27-gauge needle once every 5 days. The primary tumors and peripancreatic LNs (including pyloric, hilar, and superior mesenteric LNs) were dissected for IHC analysis at the endpoint.

### PET-CT analysis of mouse orthotopic tumors.

The nude mouse orthotopic tumors were evaluated using PET-CT. The mice were fasted for 8 hours before scanning and were anesthetized with pentobarbital. Subsequently, 5 Ci/g ^18^FDG in 50 μL 0.9% saline was injected into the tail vein. The PET-CT scanning was performed 30 minutes after the ^18^FDG injection. ^18^FDG uptake in the tumor was calculated in 3-dimensional regions.

### CRISPR/Cas9-mediated gene deletion.

A pair of sgRNAs targeting the hnRNPA1 or VEGFR3 coding sequence were cloned into the lentiCRISPR v2 (Addgene, 52961) plasmid and stably transfected into the HLECs to knock out hnRNPA1 or VEGFR3 expression (hnRNPA1^KO^ or VEGFR3^KO^). Knockout efficiency was determined using Western blotting analysis.

### Fluorescent assessment of in vitro and in vivo EV internalization.

EVs were labeled with PKH67 according to the instructions of the PKH67 green fluorescent labeling kit (Sigma-Aldrich, MINI67) and excess dye was neutralized using 5% BSA. Then, the PKH67-labeled EVs were precipitated by ultracentrifugation to yield 10 μg/mL EVs. For the in vitro assays, the EVs were incubated with HLECs for 6 hours at 37°C in 5% CO_2_. The HLECs were washed with PBS 3 times, fixed in formaldehyde for 15 minutes, and the nuclei were stained with DAPI for 5 minutes. For the in vivo assays, the EVs were injected into the footpad or pancreas of the mice every 5 days. At the endpoint of the animal experiments, the tumor tissues were dissected for analysis by immunofluorescence. The images were captured under a Zeiss confocal microscope system.

### Co-IP assay for SUMOylation modification.

HnRNPA1 SUMOylation was evaluated by co-IP assays. Cells cotransfected with His-*SUMO2* and hnRNPA1^WT^, hnRNPA1^K3R^, hnRNPA1^K113R^, or hnRNPA1^K3/K113R^ were lysed in lysis buffer containing protease inhibitors and 20 mM *N*-ethylmaleimide, and the lysates were sonicated for 1 minute. The lysates were then centrifuged at 16,000*g* for 20 minutes at 4°C. The supernatants were incubated with the respective antibodies at 4°C overnight before protein G beads were added for 2 hours. The beads were washed 3 times with cold PBS plus 0.5 M NaCl, followed by an additional wash with PBS. The immunoprecipitants were re-extracted in lysis buffer containing 1% SDS and denatured by heating for 5 minutes. The supernatants were diluted with regular lysis buffer until the concentration of SDS had decreased to 0.1%, followed by re-IP with the indicated antibodies. The immunoprecipitants were analyzed by immunoblotting with anti-His and anti-hnRNPA1 antibodies.

### Determination of the physical interactions between EV-packaged hnRNPA1 and PROX1 mRNA.

To identify the interaction between EV-packaged hnRNPA1 and *PROX1* mRNA, RIP assays were performed using an EZ-Magna RIP kit (Millipore, 17-701) according to the manufacturer’s instructions. Briefly, 2 × 10^7^ HLECs treated with 10 μg/mL EVs were harvested and lysed in cell lysis buffer containing RNase and protease inhibitors. Then, magnetic bead–coupled anti-hnRNPA1 antibodies (Abcam, ab5832) or normal rabbit IgG as the negative control were added to the cell lysate and immunoprecipitated at 4°C overnight. Next, the magnetic beads were washed with RIP washing buffer. The combined RNA was extracted for qRT-PCR analysis, in which *U1* was used as the nonspecific control. Supplemental Tables 6 and 7 list the primer sequences and the antibodies used, respectively.

### Actinomycin D–dependent mRNA stability assays.

To measure the half-life of endogenous mRNAs, we added the transcription inhibitor actinomycin D (2 μg/mL, Sigma-Aldrich) to HLECs preincubated with 10 μg/mL EVs and collected the RNA samples at 0, 6, 12, 18, and 24 hours. Then, we isolated the total RNAs with TRIzol (Life Technologies). The mRNA expression was detected by qRT-PCR and agarose gel electrophoresis. Supplemental Table 6 shows the primer sequences used.

### Bioinformatic analysis.

The SUMO2 binding site of hnRNPA1 was predicted using GPS-SUMO (54). The hnRNPA1 structural model was obtained from SWISS-MODEL (55). The *PROX1* mRNA AREs were predicted using AREsite2 (56).

### Additional methods.

Additional methods are provided in the Supplemental Methods, including plasmid construction and retroviral transduction, IHC analysis, RNA extraction and qRT-PCR assays, EV isolation and purification, electron microscopy, tube formation assays, Transwell assays, co-IP assays, Western blotting analysis, immunofluorescence, and dual-luciferase assays for the promoter and 3′-UTR activity. Supplemental Table 7 shows the antibodies used in this study.

### Statistics.

All experiments were conducted 3 or more times independently. Quantitative data are presented as the mean ± SD. The statistical difference between parametric variables was identified using a 2-tailed Student’s *t*-test or 1-way ANOVA. Nonparametric variables were compared using the χ^2^ test. The patients’ OS and DFS were evaluated using the Kaplan-Meier method. All analyses were conducted using SPSS v.13.0 (IBM). A *P* value of less than 0.05 was considered statistically significant.

### Study approval.

This study obtained the written consent of all patients and had received the approval of the Committees for Ethical Review of Research involving Human Subjects at Sun Yat-sen University and Guangdong Provincial People’s Hospital [approval number: 2013(40)]. The animal studies were performed after receiving approval from the Sun Yat-sen University Institutional Animal Care and Use Committee.

## Author contributions

CC and RC participated in the study design. Y Luo, ZL, and YK performed the in vitro and in vivo experiments. WH and YZ conducted the data analyses. HZ, Y Lin, and MA performed the clinical data analyses. DZ and JY performed the immunofluorescence and IHC experiments. Y Luo, ZL, YK, and CC wrote the manuscript. WH interpreted the data and revised the manuscript. All authors read and approved the final manuscript. The order of the co–first authors was assigned based on the relative contributions of these individuals.

## Supplementary Material

Supplemental data

## Version 1 05/17/2022

In-Press Preview

## Version 2 07/15/2022

Electronic publication
