# Supplementary material for: KRAS mutant–driven SUMOylation controls extracellular vesicle transmission to trigger lymphangiogenesis in pancreatic cancer
Source: J Clin Invest. 2022 Jul 15;132(14):e157644. doi: 10.1172/JCI157644 (PMC9282935; doi:10.1172/JCI157644)
Supplement: Supplemental data [file jci-132-157644-s016.pdf]

Supplemental Figures

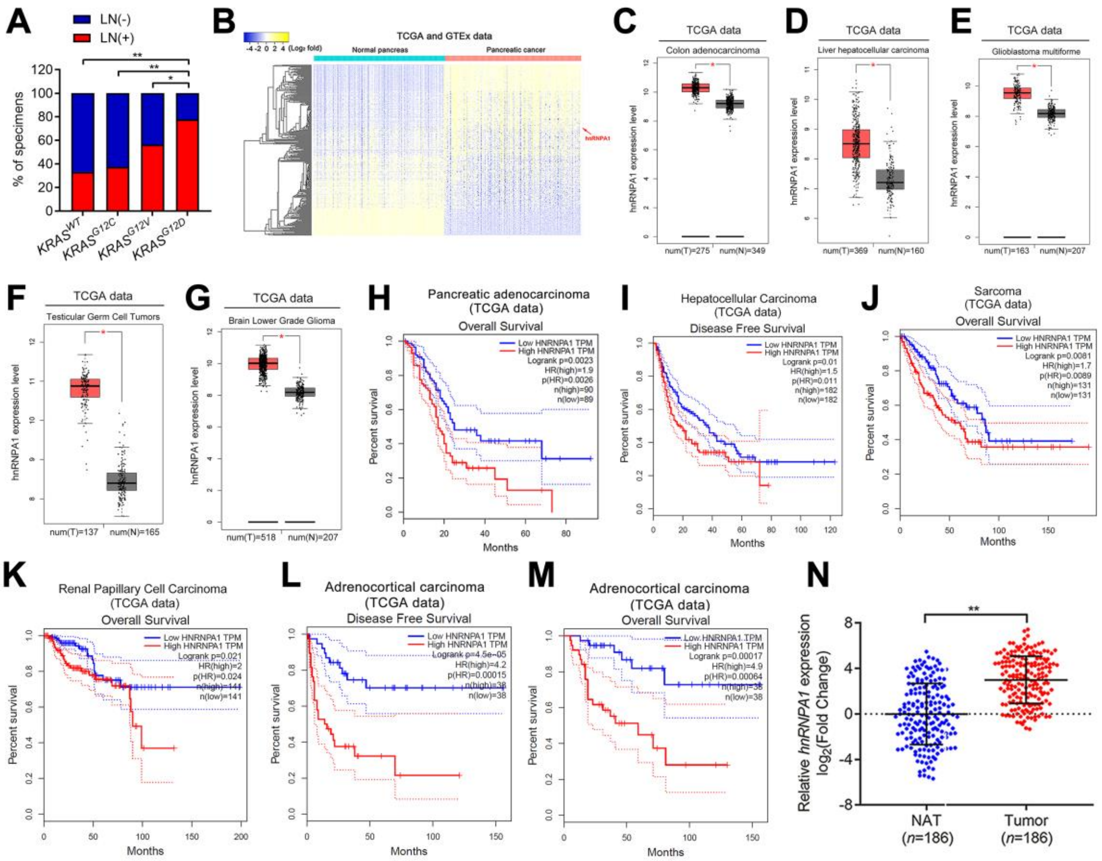

Supplemental Figure 1. HnRNPA1 is upregulated and associated with poor prognosis in human cancers. (**A**) The rate of LN metastasis in PDAC with different KRAS subtypes. The χ^2 test was used. (**B**) Unsupervised hierarchical clustering of differentially expressed genes. (**C-G**) HnRNPA1 expression in different type of cancers and corresponding normal tissues were analyzed from TCGA database by GEPIA (http://gepia.cancer-pku.cn/index.html). The nonparametric Mann-Whitney U test was used for group comparison. (**H-M**) The Kaplan-Meier curves of OS or DFS for LN-positive patients with low hnRNPA1 *vs*. high hnRNPA1 expression in multiple cancers from TCGA database by GEPIA (http://gepia.cancer-pku.cn/index.html). The median expression was set as the cut-off value. (**N**) qRT-PCR analysis of hnRNPA1 expression in PDAC tissues and paired NATs (n = 186). The nonparametric Mann-Whitney U test was used. *$p < 0.05$, **$p < 0.01$.

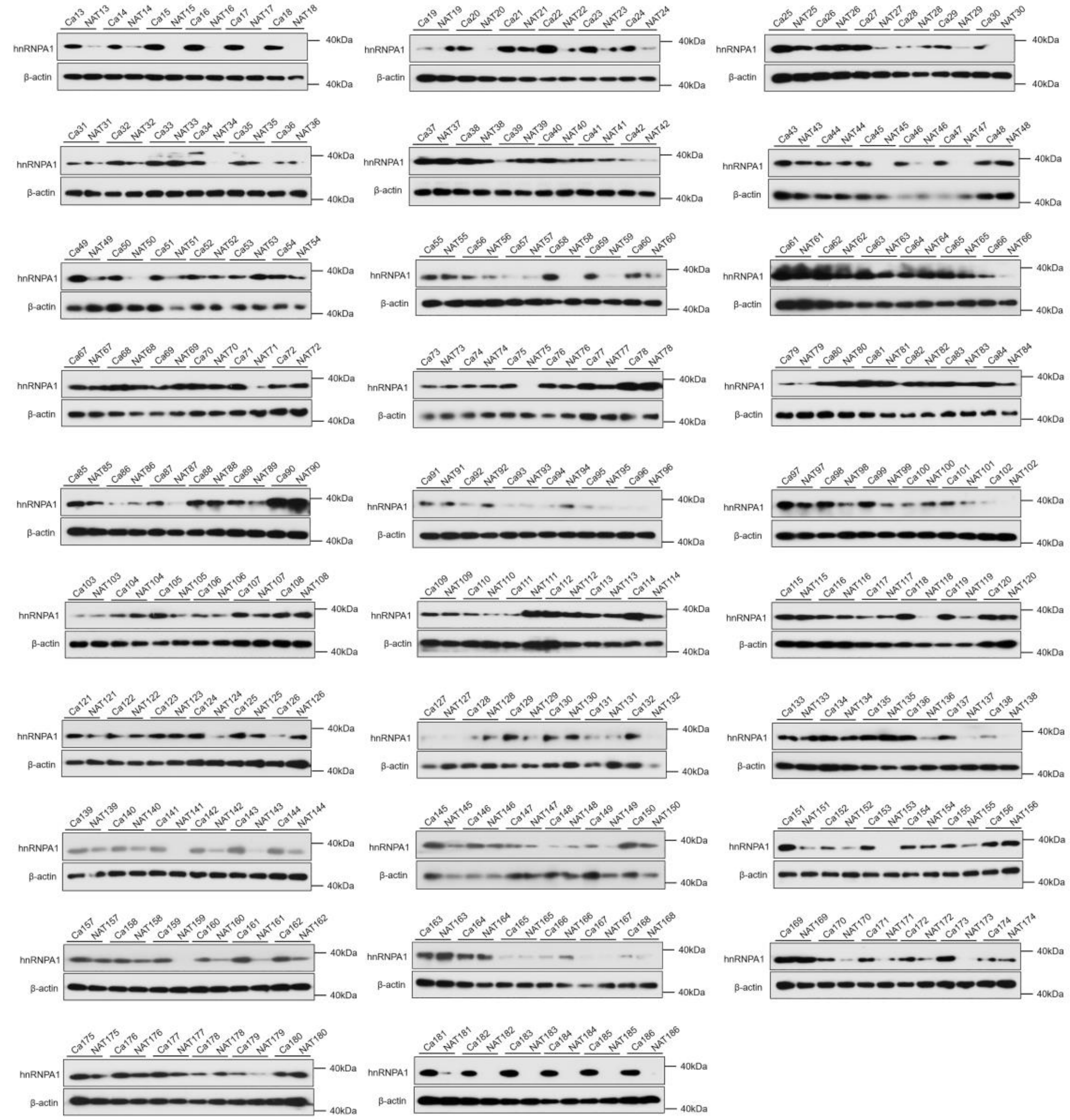

Supplemental Figure 2. The protein level of hnRNPA1 is upregulated in PDAC. Western Blotting analysis of hnRNPA1 expression in PDAC tissues and paired NATs (*n* = 186).

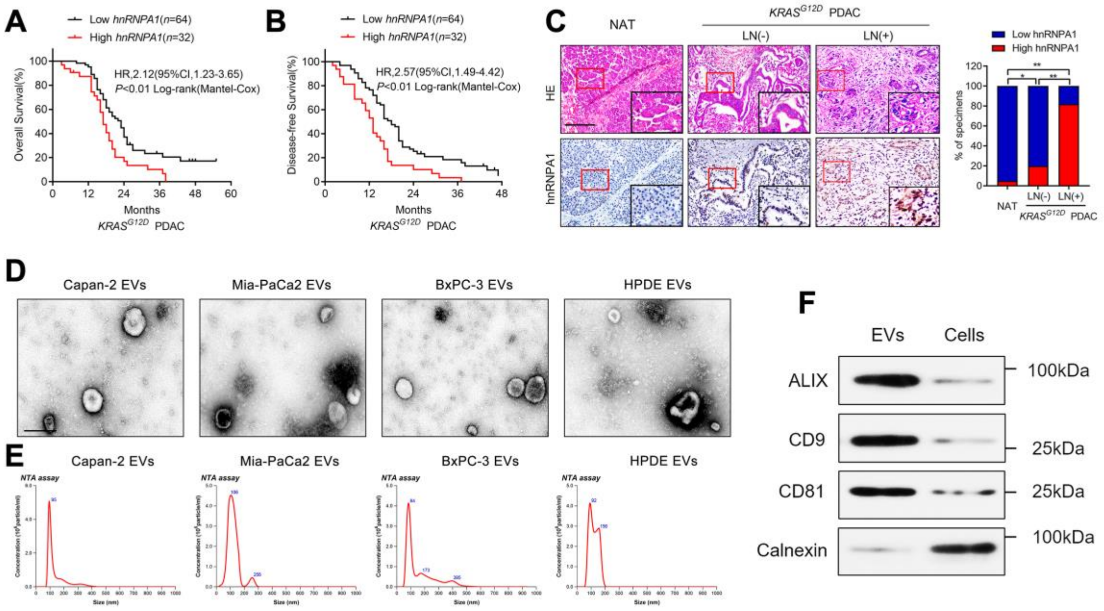

Supplemental Figure 3. HnRNPA1 is enriched in EVs secreted by *KRAS*^{G12D} PDAC. (**A** and **B)** The Kaplan-Meier curves of OS and DFS for PDAC patients with *KRAS*^{G12D} mutation according to hnRNPA1 expression. The best cut-off value was set. (**C**) Representative IHC images and percentages of hnRNPA1 expression in LN-positive or LN-negative *KRAS*^{G12D} PDAC tissues and NATs. Scale bars: 50 µm. The χ^2 test was used. (**D** and **E**) TEM- and NanoSight-characterized EVs secreted by *KRAS*^{G12C}, *KRAS*^{G12V}, *KRAS*^{WT} PDAC cells and HPDE cells. Scale bar: 100 nm. (**F**) Western Blotting analysis of EVs and cellular markers in PDAC cells and corresponding EVs. *$p < 0.05$, **$p < 0.01$.

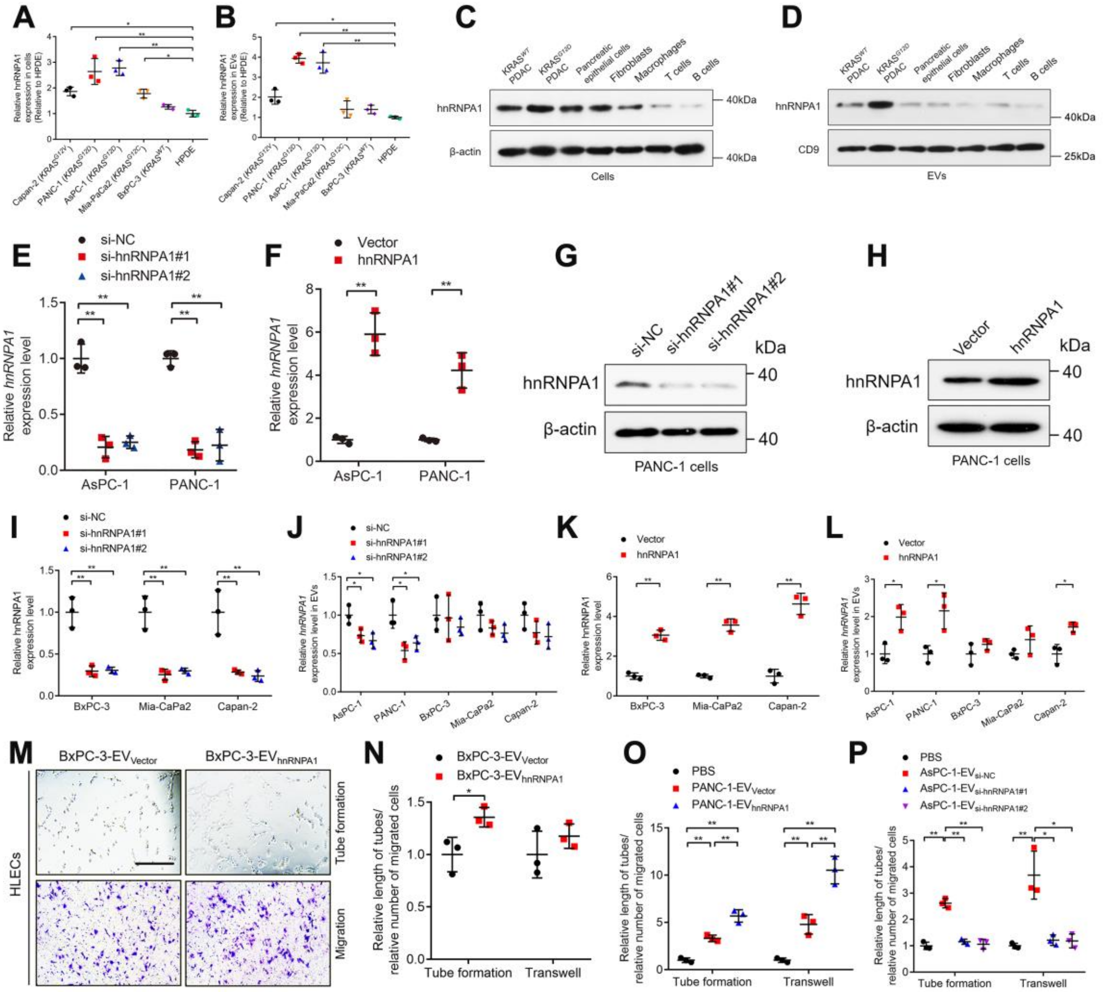

Supplemental Figure 4. *KRAS*^{G12D} PDAC-secreted EV-packaged hnRNPA1 induces lymphangiogenesis *in vitro*. (**A** and **B**) qRT-PCR analysis of hnRNPA1 expression in PDAC cells with different *KRAS* subtypes and the corresponding EVs. ANOVA followed by Dunnett's tests were used. (**C** and **D**) Western Blotting analysis of hnRNPA1 expression in *KRAS*^{G12D} PDAC cells, pancreatic epithelial cells, fibroblasts, macrophages, T cells, B cells and corresponding EVs. (**E** and **F**) qRT-PCR of hnRNPA1 expression levels in indicated *KRAS*^{G12D} PDAC cells. 2-tailed Student's *t*-test or ANOVA followed by Dunnett's tests were used. (**G** and **H**) Western Blotting analysis of hnRNPA1 protein levels in PANC-1 cells after hnRNPA1 silencing or overexpression. (**I-L**) qRT-PCR of hnRNPA1 expression in indicated PDAC cells and corresponding EVs. 2-tailed Student's *t*-test or ANOVA followed by Dunnett's tests were used. (**M-N**) Representative images and quantification of tube formation and Transwell migration for BxPC-3-EV$_{Vector}$ or BxPC-3-EV$_{hnRNPA1}$-treated HLECs. 2-tailed Student's *t*-test were used. (**O-P**) Quantification of tube formation and Transwell migration

for PBS, PANC-1-EV_{Vector}, PANC-1-$EV_{hnRNPA1}$ or AsPC-1-$EV_{si\text{-}NC}$, AsPC-1-$EV_{si\text{-}hnRNPA1\#1}$, or AsPC-1-$EV_{si\text{-}hnRNPA1\#2}$-treated HLECs. ANOVA followed by Dunnett's tests were used. Figures with error bars show the SD of three independent experiments. *$p < 0.05$, **$p < 0.01$.

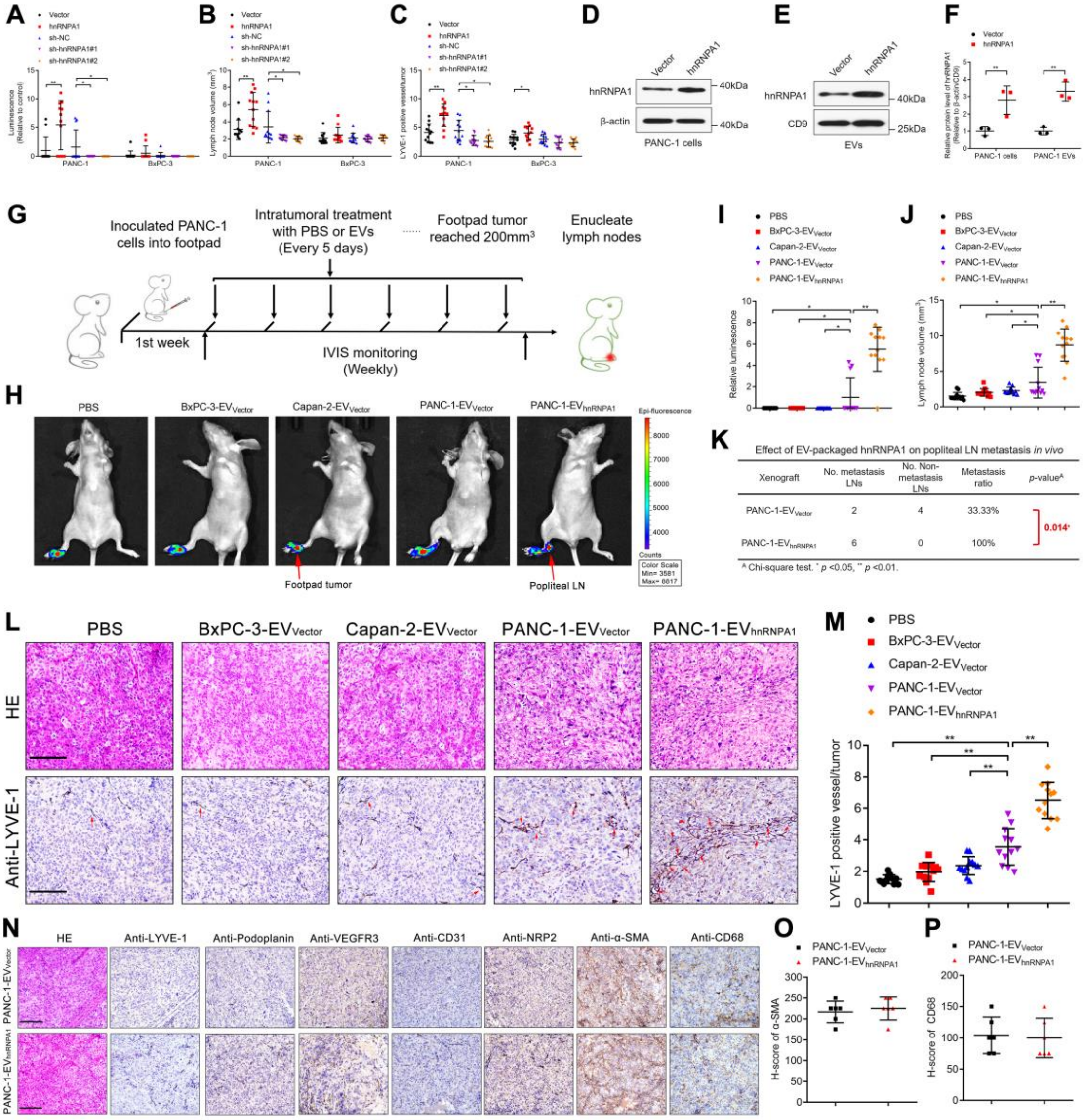

Supplemental Figure 5. EV-packaged hnRNPA1 promotes lymphangiogenesis and LN metastasis of *KRAS*^{G12D} PDAC in popliteal LN metastasis model. (**A**) Quantification of bioluminescence of the popliteal metastatic LNs in indicated groups ($n = 12$ per group). ANOVA followed by Dunnett's tests were used. (**B**) Quantification of the popliteal LN volume in indicated groups ($n = 12$ per group). ANOVA followed by Dunnett's tests were used. (**C**) Quantification of LYVE-1-marked lymphatic vessel density in footpad tumors ($n = 12$ per group). ANOVA followed by Dunnett's tests were used. (**D-F**) Western Blotting

analysis and quantification of hnRNPA1 expression in PANC-1 cells and corresponding EVs after hnRNPA1 overexpression. 2-tailed Student's *t*-test were used. (**G**) Schematic representation of the establishment of the popliteal lymphatic metastasis model. (**H** and **I**) Representative images and quantification of bioluminescence of the popliteal metastatic LNs after treatment with indicated EVs (n = 12 per group). Red arrows: Footpad tumor and metastatic popliteal LN. ANOVA followed by Dunnett's tests were used. (**J**) Quantification of the popliteal LN volume after treatment with indicated EVs (n = 12 per group). ANOVA followed by Dunnett's tests were used. (**K**) The LN metastasis rate in indicated groups of EV-induced popliteal LN metastasis model. The χ^2 test was used. (**L** and **M**) Representative IHC images and quantification of LYVE-1-marked lymphatic vessel density in footpad tumors after treatment with indicated EVs (n = 12 per group). Scale bars: 50 μm. ANOVA followed by Dunnett's tests were used. (**N-P**) Representative IHC images of LYVE-1-, Podoplanin-, VEGFR3-, CD31- or NRP2-marked lymphatic vessel density, α-SMA-marked CAFs and CD68-marked TAMs in footpad tumors (n = 6 per group). Scale bars: 50 μm. Figures with error bars show the SD of three independent experiments. *$p < 0.05$, **$p < 0.01$.

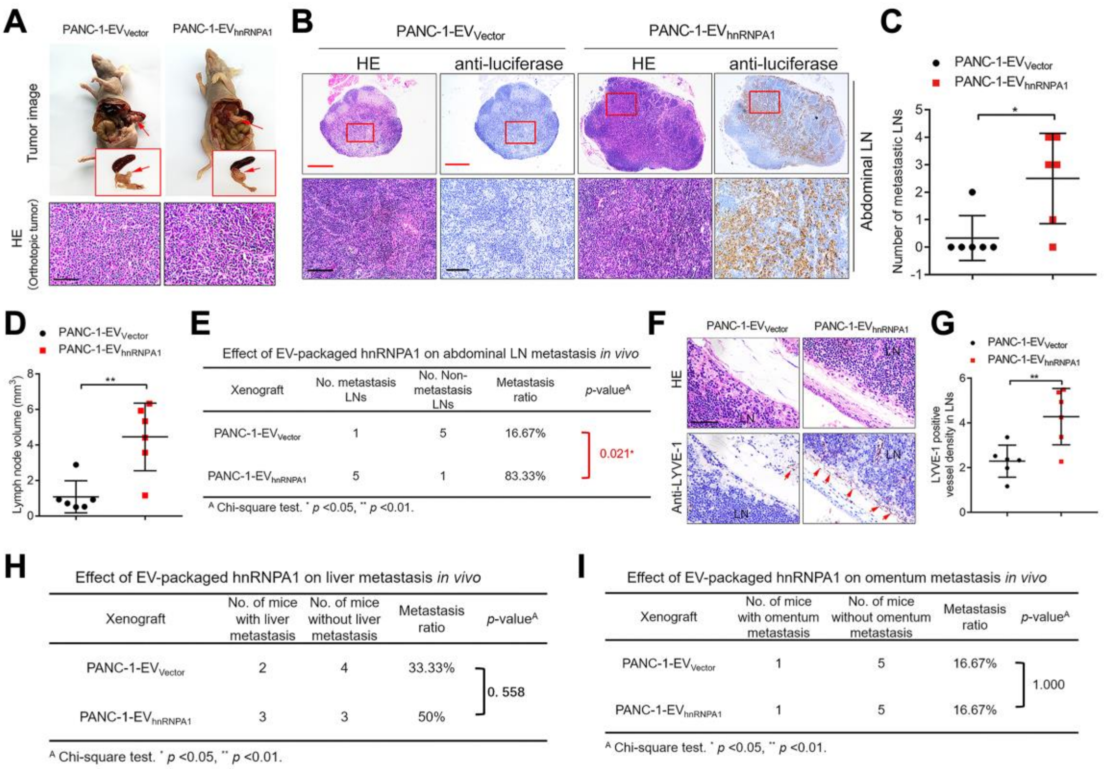

Supplemental Figure 6. EV-packaged hnRNPA1 triggers lymphangiogenesis and LN metastasis of *KRAS*^{G12D} PDAC in orthotopic xenograft model. (**A**) Representative images and HE staining for orthotopic tumors. Scale bars: 50 µm. Red arrow: orthotopic tumor. (**B**) Representative images of HE and IHC staining with anti-luciferase antibody of peripancreatic LNs. Scale bars: 500 µm (red) or 50 µm (black). 2-tailed Student's *t*-tests were used. (**C** and **D**) Quantification of the metastatic number (**C**) and average volume (**D**) of peripancreatic LNs (n = 6 per group). 2-tailed Student's *t*-tests were used. (**E**) The LN metastasis rate in indicated groups of orthotopic xenograft model. The χ^2 test was used. (**F** and **G**) Representative IHC images and quantification of LYVE-1-marked lymphatic vessel density in subcapsular sinus of peripancreatic LNs (n = 6). 2-tailed Student's *t*-tests were used. (**H** and **I**) The liver and omentum metastasis rate in indicated groups of orthotopic xenograft model. The χ^2 test was used. Figures with error bars show the SD of three independent experiments. $*p < 0.05$, $**p < 0.01$.

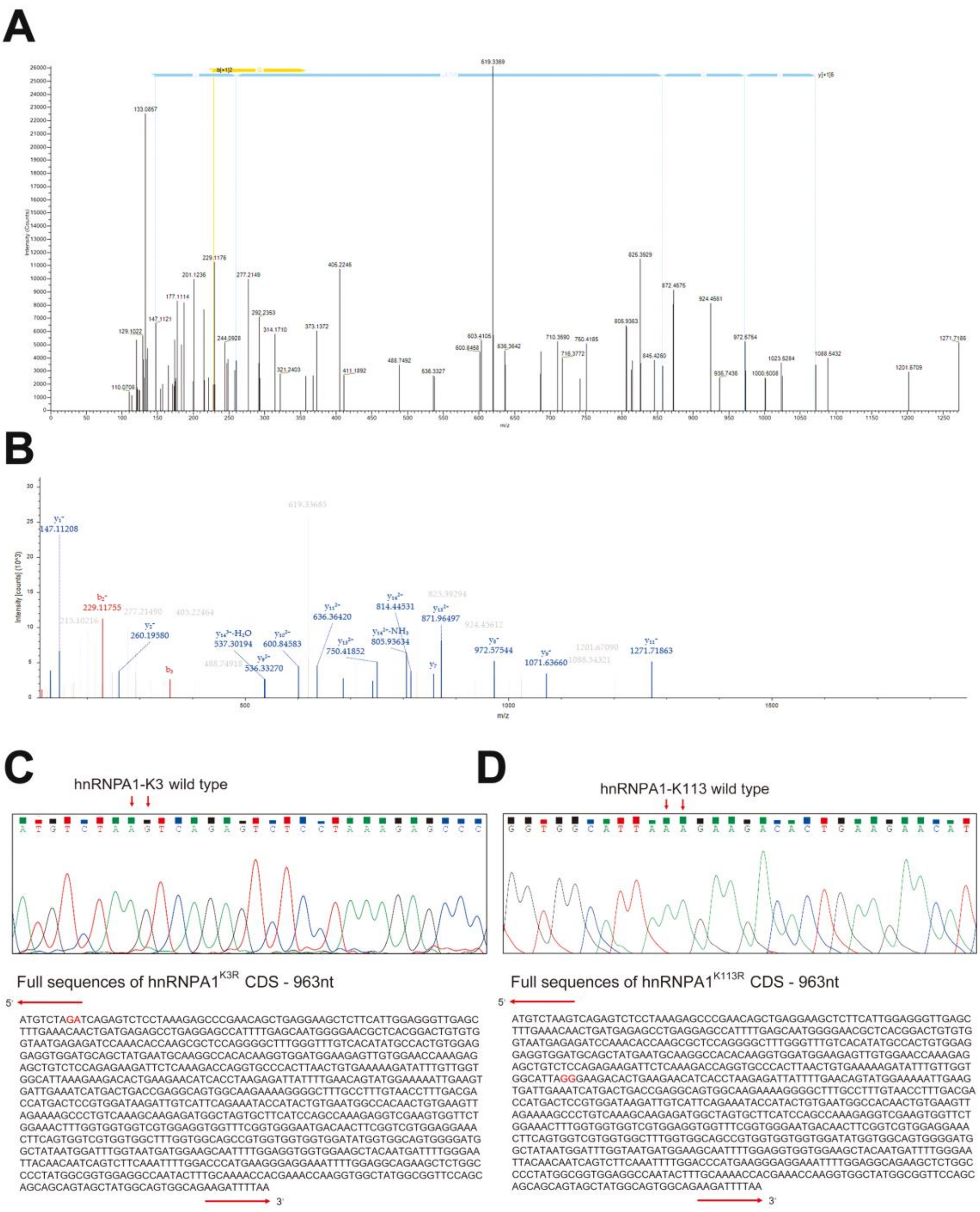

Supplemental Figure 7. HnRNPA1 is SUMOylated on K113 residue. (**A-B**) The hnRNPA1 Co-IP products were performed with mass septectomy (MS) to identify SUMO2 protein. (**C-D**) Sequencing evaluated the wild type of K3 and K113 residue on hnRNPA1. The full sequences of hnRNPA1^{K3R} or hnRNPA1^{K113R} coding sequences (CDS) were shown.

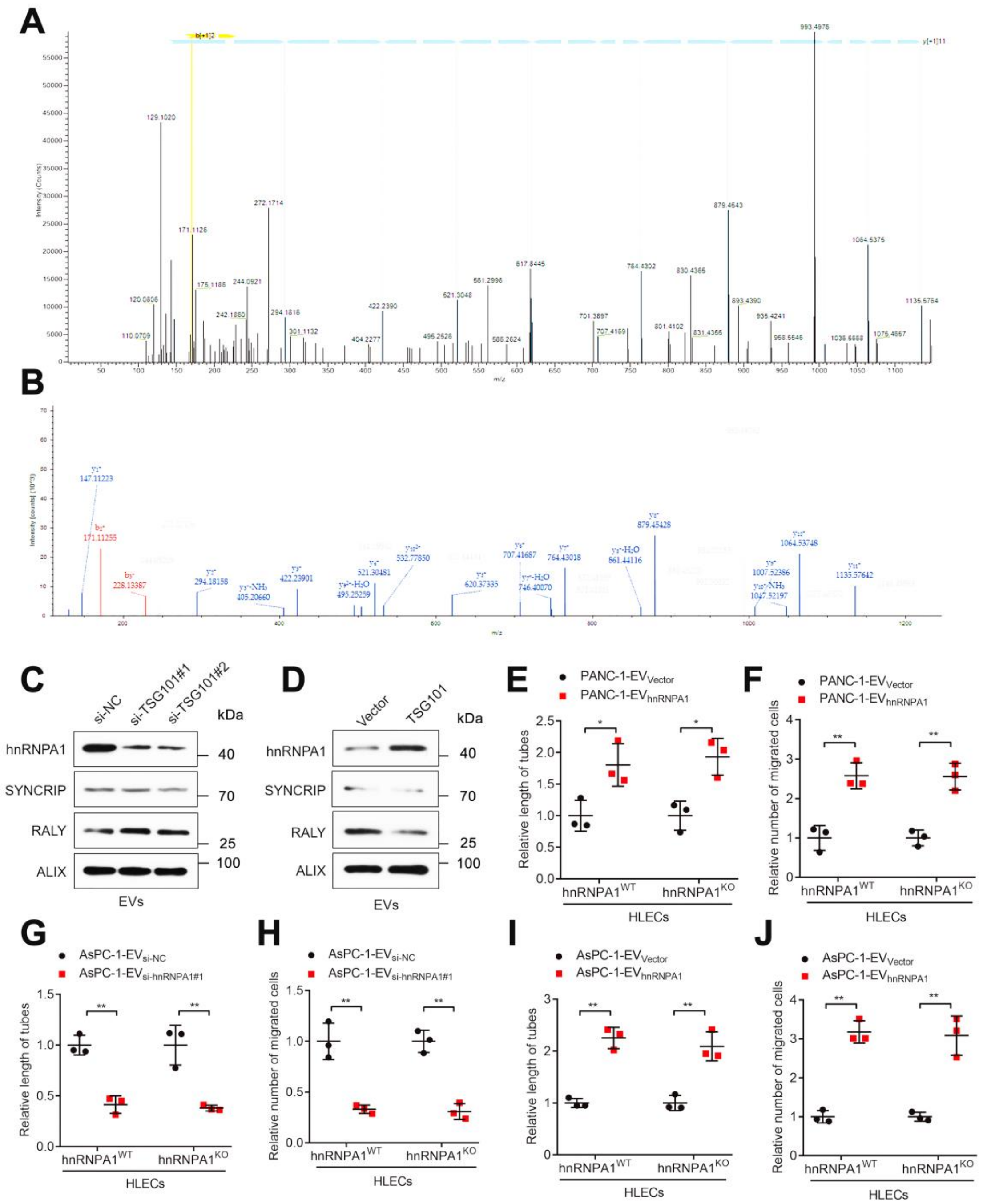

Supplemental Figure 8. SUMOylated hnRNPA1 is packaged into EVs through the interaction with TSG101. (**A-B**) MS assay identified that hnRNPA1 specifically binds to TSG101. (**C** and **D**) Western Blotting of indicated proteins expression in PANC-1 cells after TSG101 knockdown or overexpression. (**E-J**) Quantification of tube formation and Transwell migration for PANC-1-EV$_{Vector}$, PANC-1-EV$_{hnRNPA1}$, AsPC-1-EV$_{si\text{-}NC}$, AsPC-1-EV$_{si\text{-}hnRNPA1\#1}$ or AsPC-1-EV$_{Vector}$, AsPC-1-EV$_{hnRNPA1}$-treated hnRNPA1^{WT} and hnRNPA1^{KO} HLECs groups.

2-tailed Student’s *t*-test were used. Figures with error bars show the SD of three independent experiments. *$p < 0.05$, **$p < 0.01$.

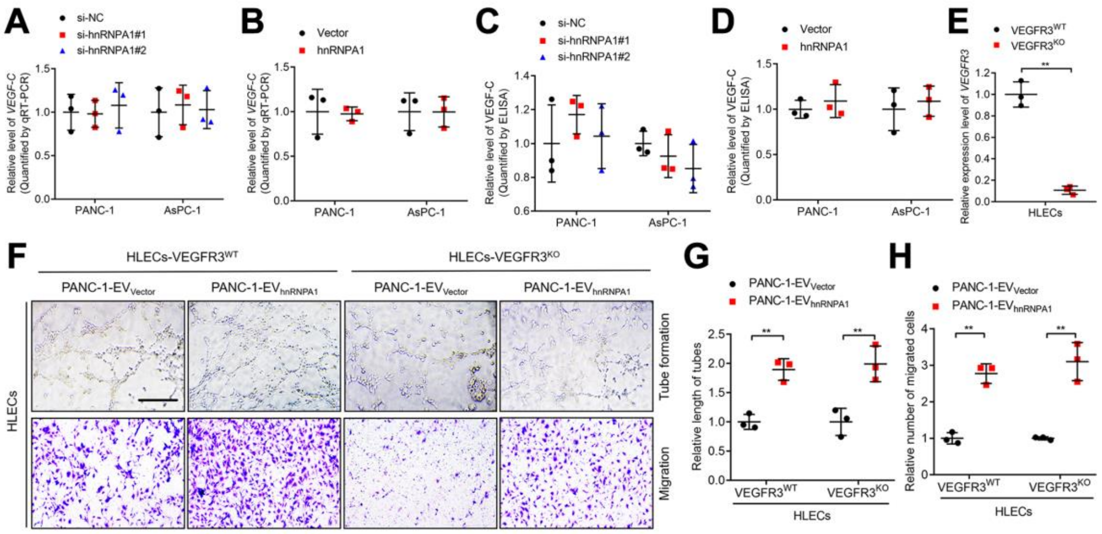

Supplemental Figure 9. EV-packaged hnRNPA1 induces lymphangiogenesis in a VEGF-C independent manner. (**A-D**) qRT-PCR and ELISA analysis of VEGF-C expression and secretion in PDAC cells after downregulating or overexpressing hnRNPA1. 2-tailed Student’s *t*-test or ANOVA followed by Dunnett’s tests were used. (**E**) qRT-PCR validation of VEGFR3 knockout in HLECs. (**F-H**) Representative images and quantification of tube formation and Transwell migration by EV-treated $VEGFR3^{WT}$ or $VEGFR3^{KO}$ HLECs. Scale bars: 100 μm. 2-tailed Student’s *t*-tests were used. Figures with error bars show the SD of three independent experiments. *$p < 0.05$, **$p < 0.01$.

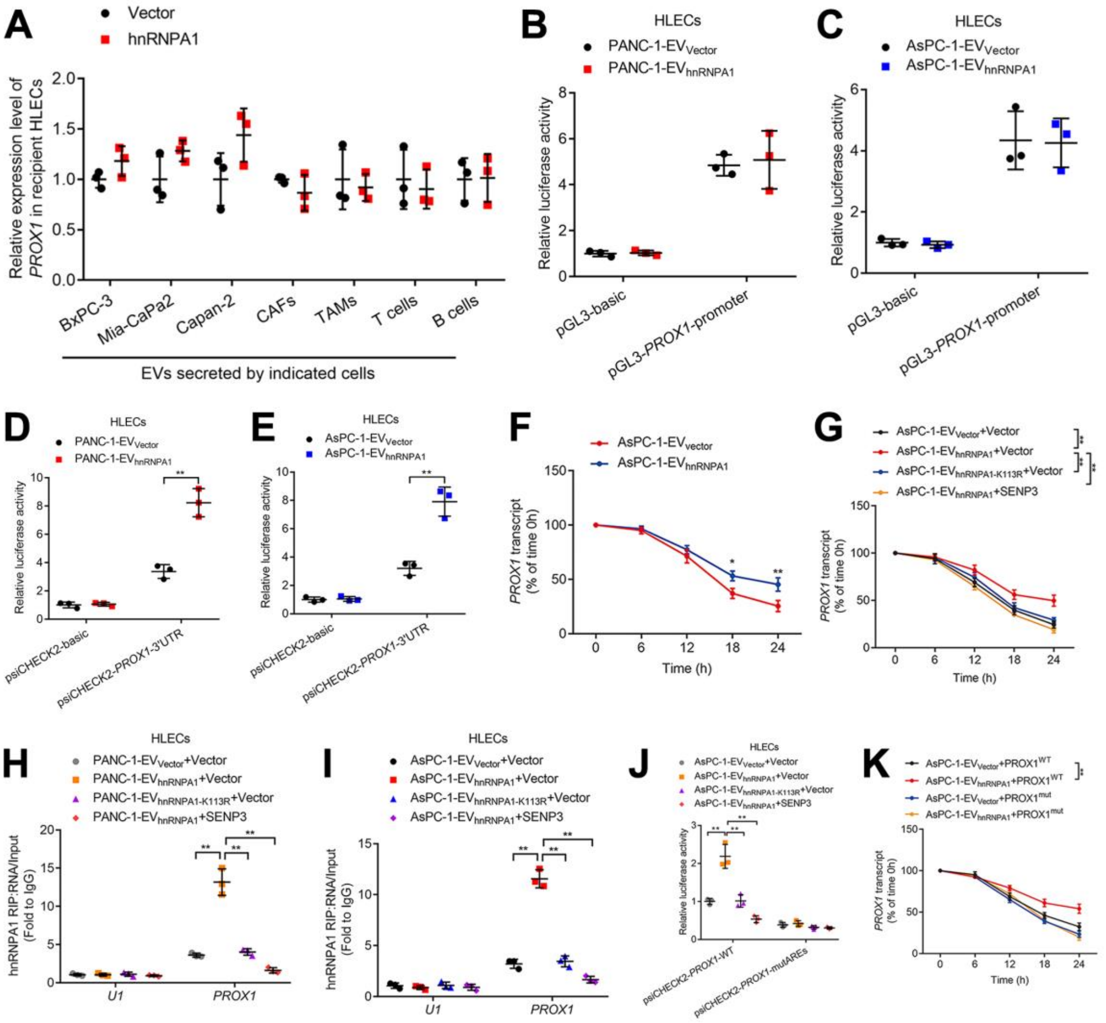

Supplemental Figure 10. EV-packaged hnRNPA1 stabilizes the PROX1 mRNA in HLECs. (**A**) qRT-PCR analysis of PROX1 expression in indicated EV-treated HLECs. 2-tailed Student's *t*-test were used. (**B-E**) Dual luciferase assays for the promoter or 3'UTR of PROX1 in indicated HLECs. 2-tailed Student's *t*-test were used. (**F**) Actinomycin assays for PROX1 mRNA in AsPC-1-EV$_{Vector}$ and AsPC-1-EV$_{hnRNPA1}$-treated HLECs. (**G**) Actinomycin assays for PROX1 mRNA in AsPC-1-EV$_{Vector}$, AsPC-1-EV$_{hnRNPA1}$, AsPC-1-EV$_{hnRNPA1-K113R}$-treated HLECs with or without SENP3 overexpression. (**H** and **I**) RIP of hnRNPA1-associated RNAs in HLECs. ANOVA followed by Dunnett's tests were used. (**J**) Dual-luciferase assays of wild-type or ARE-mutated PROX1 in indicated AsPC-1 EV-treated HLECs. ANOVA followed by Dunnett's tests were used. (**K**) Actinomycin assays for PROX1 mRNA in AsPC-1-EV$_{Vector}$, AsPC-1-EV$_{hnRNPA1}$-treated HLECs with or without AREs mutation on PROX1. Figures with error bars show the SD of three independent experiments. *$p < 0.05$, **$p < 0.01$.

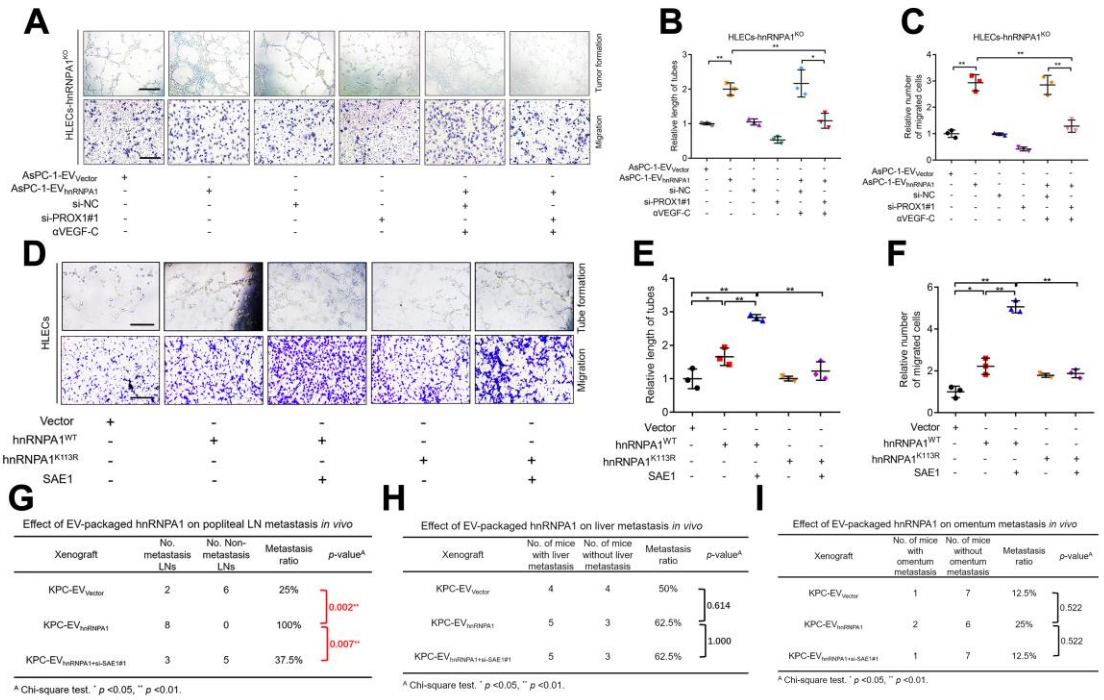

Supplemental Figure 11. PROX1 is required for EV-packaged hnRNPA1-mediated lymphangiogenesis. (**A-C**) Representative images and quantification of tube formation and Transwell migration by EV-treated hnRNPA1^{KO} HLECs with or without PROX1 knockdown, and VEGF-C neutralizing antibody. Scale bars: 100 μm. ANOVA followed by Dunnett's tests were used. (**D-F**) Representative images and quantification of tube formation and Transwell migration by HLECs with or without hnRNPA1^{WT}, hnRNPA1^{K113R} or SAE1 overexpression. Scale bars: 100 μm. ANOVA followed by Dunnett's tests were used. (**G-I**) The LN, liver or omentum metastasis rate in indicated groups of KPC mice model. The χ^2 test was used. Figures with error bars show the SD of three independent experiments. *$p < 0.05$, **$p < 0.01$.

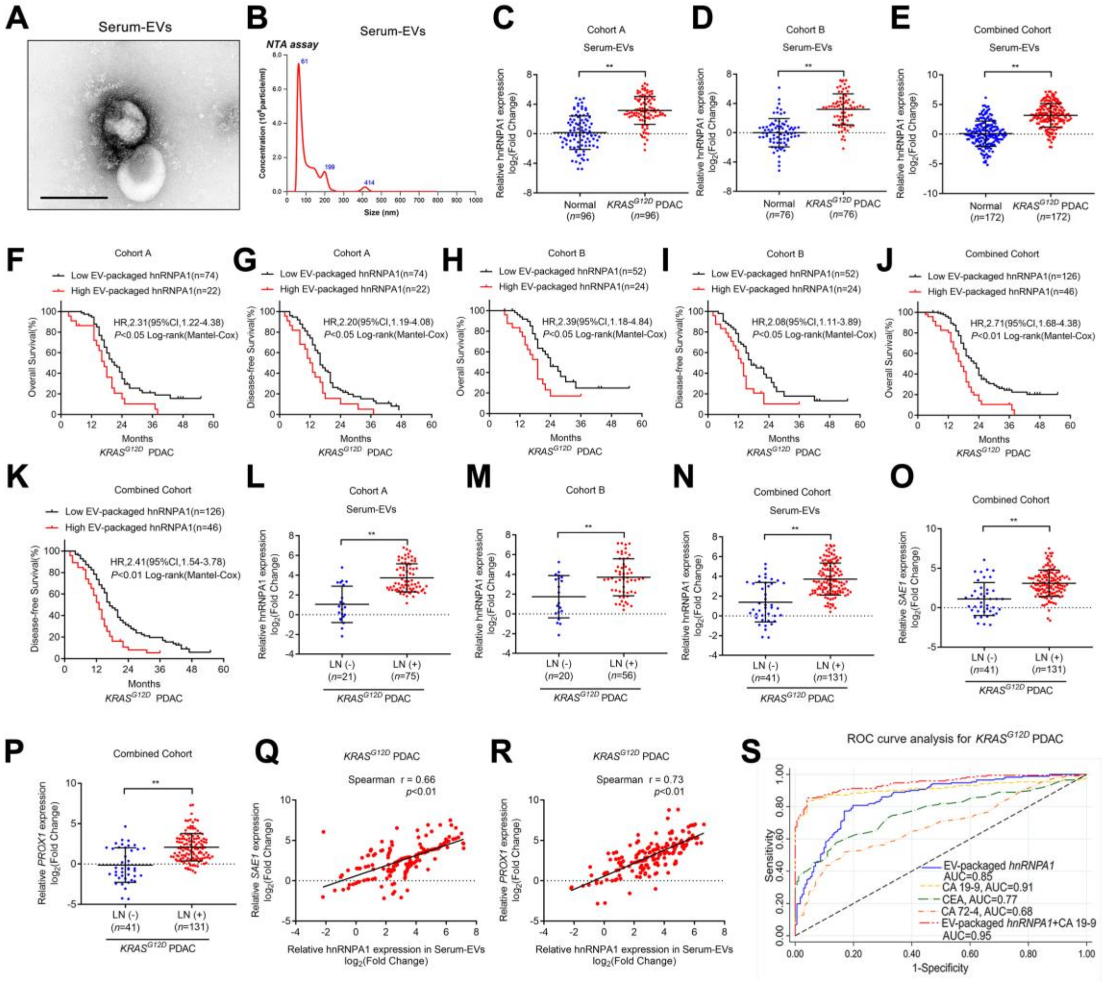

Supplemental Figure 12. EV-packaged hnRNPA1 is associated with LN metastasis in *KRAS*^{G12D} PDAC. (**A**-**B**) TEM- and NanoSight-characterized serum-EVs obtained from BCa patients. Scale bar: 100 nm. (**C-E**) qRT-PCR analysis of hnRNPA1 expression in serum-EVs obtained from *KRAS*^{G12D} PDAC patients and healthy participants in two independent cohorts and the combined cohort. The nonparametric Mann-Whitney U test was used. (**F-K**) The Kaplan-Meier curves of OS or DFS for *KRAS*^{G12D} PDAC patients according to EV-packaged hnRNPA1 expression in two independent cohorts and the combined cohort. The best cut-off value was set. (**L-N**) qRT-PCR analysis of hnRNPA1 expression in serum-EVs obtained from *KRAS*^{G12D} PDAC patients according to LN status in two independent cohorts and the combined cohort. The nonparametric Mann-Whitney U test was used. (**O-P**) qRT-PCR analysis of SAE1 and PROX1 expression in serum-EVs obtained from *KRAS*^{G12D} PDAC patients according to LN status in the combined cohorts. The nonparametric Mann-Whitney U test was used. (**Q-R**) Correlation analysis between EV-packaged

hnRNPA1 and SAE1 or PROX1 expression. (**S**) ROC analysis for the diagnostic efficiency of serum EV-packaged hnRNPA1, CA19-9, CEA and CA72-4 for *KRAS*^{G12D} PDAC. Figures with error bars show the SD. *$p < 0.05$, **$p < 0.01$.

Supplemental Tables

Supplemental Table 1. The upregulated hnRNPs identified from TCGA and GTEx dataset

Gene	Overall survival		Disease-free survival		PDAC vs. Non-tumorous tissues
	P-value	HR	P-value	HR	P-value
hnRNPA1	**0.002**	**1.9**	**0.043**	**1.6**	9.97×10^{-05}
RALY	**0.030**	**1.6**	**0.043**	**1.6**	9.74×10^{-87}
SYNCRIP	**0.047**	**1.5**	**0.007**	**1.8**	1.55×10^{-24}
hnRNPC	**0.006**	**1.8**	0.110	1.4	1.54×10^{-50}
hnRNPL	**0.023**	**1.6**	0.730	1.1	2.95×10^{-43}
hnRNPA0	0.057	0.67	0.040	0.63	4.02×10^{-74}
PTBP1	0.072	1.5	0.110	1.4	1.79×10^{-54}
hnRNPD	0.120	1.4	0.110	1.4	4.27×10^{-42}
hnRNPF	0.150	1.4	0.440	1.2	1.33×10^{-65}
hnRNPK	0.160	1.3	0.280	1.3	5.18×10^{-57}
hnRNPLL	0.270	1.3	0.160	1.4	8.37×10^{-50}
PCBP1	0.290	1.2	0.460	1.2	1.8×10^{-71}
hnRNPAB	0.300	1.2	0.240	1.3	1.07×10^{-95}
hnRNPR	0.470	1.2	0.320	1.2	1.88×10^{-57}
hnRNPM	0.470	0.86	0.610	0.89	3.86×10^{-45}
hnRNPU	0.490	1.2	0.290	1.3	1.18×10^{-17}
hnRNPA3	0.560	1.1	0.280	1.3	2.18×10^{-08}
RBMX2	0.580	0.89	0.100	0.69	5.37×10^{-42}
FUS	0.620	0.91	0.082	0.68	3.52×10^{-85}
hnRNPUL1	0.640	1.1	0.670	1.1	2.39×10^{-60}
hnRNPA2B1	0.680	1.1	0.340	1.2	1.42×10^{-17}
hnRNPH2	0.860	1	0.670	1.1	2.23×10^{-48}
hnRNPH3	0.970	0.99	0.820	0.95	9.54×10^{-12}

Supplemental Table 2. The status of LNs in orthotopic xenograft model

mouse	Group	No. of metastatic LNs	No. of non-metastatic LNs	Total No. of enucleate LNs
1	PANC-1-EV$_{Vector}$	0	6	6
2	PANC-1-EV$_{Vector}$	0	6	6
3	PANC-1-EV$_{Vector}$	2	4	6
4	PANC-1-EV$_{Vector}$	0	6	6
5	PANC-1-EV$_{Vector}$	0	6	6
6	PANC-1-EV$_{Vector}$	0	6	6
7	PANC-1-EV$_{hnRNPA1}$	1	5	6
8	PANC-1-EV$_{hnRNPA1}$	0	6	6
9	PANC-1-EV$_{hnRNPA1}$	4	2	6
10	PANC-1-EV$_{hnRNPA1}$	3	3	6
11	PANC-1-EV$_{hnRNPA1}$	3	3	6
12	PANC-1-EV$_{hnRNPA1}$	4	2	6

Supplemental Table 3. Univariate and multivariate analysis of OS in *KRAS*^{G12D} PDAC patients (*n*=172)

Variables	Univariate analysis			Multivariate analysis		
	**HR**	**95%CI**	***p*-Value[a]**	**HR**	**95%CI**	***p*-Value[a]**
Age (>60 vs. ≤60)	1.199	0.824-1.771	0.377			
Gender (male vs. female)	0.912	0.643-1.354	0.783			
Differentiation (moderately or poor vs. well)	1.101	0.791-1.561	0.653			
T stage (T3-4 vs. T1-2)	0.921	0.621-1.283	0.650			
TNM stage (AJCC) (stage II or stage III vs. stage I)	1.613	0.984-2.412	0.059			
Lymphatic metastasis (positive vs. negative)	1.711	1.217-2.601	**0.003****	1.572	0.901-2.612	0.104
EV-packaged hnRNPA1 expression (high vs. low)	1.812	1.323-2.399	**0.002****	1.623	1.089-2.015	**0.018***

Abbreviations: HR = hazard ratio; 95%CI = 95% confidence interval; [a] Cox regression analysis, * $p<0.05$, ** $p<0.01$.

Supplemental Table 4. Univariate and multivariate analysis of DFS in *KRAS*^{G12D} PDAC patients (*n*=172)

Variables	Univariate analysis			Multivariate analysis		
	**HR**	**95%CI**	***p*-Value[a]**	**HR**	**95%CI**	***p*-Value[a]**
Age (>60 vs. ≤60)	1.177	0.891-1.821	0.695			
Gender (male vs. female)	0.933	0.671-1217	0.899			
Differentiation (moderately or poor vs. well)	0.917	0.628-1.453	0.757			
T stage (T3-4 vs. T1-2)	1.055	0.770-1.446	0.740			
TNM stage (AJCC) (stage II or stage III vs. stage I)	1.466	1.076-2.311	0.052			
Lymphatic metastasis (positive vs. negative)	1.754	1.211-2.398	**0.006****	1.511	0.961-2.521	0.063
EV-packaged hnRNPA1 expression (high vs. low)	1.799	1.237-2.358	**0.002****	1.597	1.079-2.112	**0.021***

Abbreviations: HR = hazard ratio; 95%CI = 95% confidence interval. [a] Cox regression analysis, $^{*}p<0.05$, $^{**}p<0.01$.

Supplemental Table 5. Correlation between EV-packaged hnRNPA1 expression and clinicopathologic characteristics of *KRAS*^{G12D} PDAC patients

Characteristics	No. of cases	EV-packaged hnRNPA1 expression level		
		Low	High	*p*-Value[a]
**Total cases**	172	86	86	
**Gender**				0.644
Male	97	50	47	
Female	75	36	39	
**Age**				0.877
≤60	67	34	33	
＞60	105	52	53	
**Differentiation**				0.729
Poor	33	15	18	
Moderate	107	56	51	
Well	32	15	17	
**T stage**				0.280
T1-2	99	53	46	
T3-4	73	33	40	
**TNM stage**				**0.003**[**]
Stage I	38	28	10	
Stage II	90	41	49	
Stage III	44	17	27	
**Lymphatic metastasis**				**0.001**[**]
Negative	41	31	10	
Positive	131	55	76	

Abbreviations: No. of cases = Number of cases; [a] Chi-square test, [*] $p < 0.05$, [**] $p < 0.01$.

Supplemental Table 6. Primer and probes used in the experiments.

Gene	Sequence (5’-3’)	Application
TSG101	F: GGCTACTGGACACATACCCA<br>R: GGACGAGAGAAGACTGGAGG	qRT-PCR
PROX1	F: CAGCCCGAAAAGAACAGAAG<br>R: GGGTCTAGCTCGCACATCTC	qRT-PCR
hnRNPA1	F: TCCATTATAGCCATCCCCACT<br>R: GAAAAGCCCTGTCAAAGCAAG	qRT-PCR
GAPDH	F: CATGAGAAGTATGACAACAGCCT<br>R: AGTCCTTCCACGATACCAAAGT	qRT-PCR
SAE1	F: GGTGGCTGTCTTTGTTCCAG<br>R: AGAAGGTGACAAGAGGCTCC	qRT-PCR
UBA2	F: GCTGCCCGAAACCATGTTAA<br>R: AGGAAAGGTTCTCTGGGTCG	qRT-PCR
UBC9	F: AATTCTTCGTCCTGAGGCCA<br>R: GCTTCCCATCTCTGTCCACT	qRT-PCR
SENP1	F: ACAGCCAGAGACCTTGGAAA<br>R: GATGTCCCTCACCCCTTTCA	qRT-PCR
SENP2	F: TGGCTGGTTAGGATTCTCGG<br>R: TGGTCAGCTGGAATGGGAAT	qRT-PCR
SENP3	F: AAATGAATGTGGCCAGGCAG<br>R: TGAGTTTGCAGTGACACAGC	qRT-PCR
SENP5	F: CAGTTGCAGCCCATTTCCTT<br>R: GTTGGTCAGGGAATGCTTCG	qRT-PCR
SENP6	F: TGGAAAAGTAGAAGCAGCGC<br>R: ACGACGTTTCAGAGGTGTGT	qRT-PCR
SENP7	F: TCGTCTCACTGGTATCTCGC<br>R: TGGGAATCCTCTGCACTCAA	qRT-PCR
VEGF-C	F: CTCTCTCTCAAGGCCCCAAA<br>R: AGTCATCTCCAGCATCCGAG	qRT-PCR
VEGFR3	F: GGAGAAGCTGGTCCTGAACT<br>R: ACGTTGTGGATGGTCAGGAT	qRT-PCR
U1	F: GGGAGATACCATGATCACGAAGGT<br>R: CCACAAATTATGCAGTCGAGTTTC	RIP
PROX1	F: CAGCCCGAAAAGAACAGAAG<br>R: GGGTCTAGCTCGCACATCTC	RIP
hnRNPA1-sgRNA1	CTAAAGAGCCCGAACAGCTG	CRISPR/Cas9

hnRNPA1-sgRNA2	GGCTTTGGTGGCAGCCGTGG	CRISPR/Cas9
VEGFR3-sgRNA1	CGGGTCGGACCCACGCGCAG	CRISPR/Cas9
VEGFR3-sgRNA2	AGAGGTTTTGTTCGTATTAT	CRISPR/Cas9
si-hnRNPA1#1	sense: CAGCUGAGGAAGCUCUUCATT<br>antisense: UGAAGAGCUUCCUCAGCUGTT	siRNA
si-hnRNPA1#2	Mixed:<br>rCrArArCrUrUrCrGrGrUrCrGrUrGrGrArGrGrAdTdT	siRNA
si-TSG101#1	sense: GCAGUUCCAGGGAACUAAUTT<br>antisense: AUUAGUUCCCUGGAACUGCTT	siRNA
si-TSG101#2	sense: CCCAGUAGGGAUGGCACAATT<br>antisense: UUGUGCCAUCCCUACUGGGTT	siRNA
si-PROX1#1	sense: UGGAGAAGUAUGCGCGUCATT<br>antisense: UGACGCGCAUACUUCUCCATT	siRNA
si-SAE1#1	sense: AGACAACGAUGGUCAAAAATT<br>antisense: UUUUUGACCAUCGUUGUCUTT	siRNA
si-SUMO2	sense: GCUGCUUGUGUGCUCGUUUTT<br>antisense: AAACGAGCACACAAGCAGCTT	siRNA

Supplemental Table 7. Antibodies used in the experiments.

Product	Source	No. of Catalogue
**Primary antibody:**		
Western Blotting:		
anti-β-actin	Sigma-Aldrich	A5441
anti-hnRNPA1	Abcam	ab5832
anti-RALY	Abcam	ab170105
anti-SYNCRIP	Abcam	ab184946
anti-TSG101	Abcam	ab83
anti-CD9	Cell Signaling Technology	13403
anti-CD81	Abcam	ab79559
anti-Calnexin	Abcam	ab133615
anti-ALIX	Cell Signaling Technology	92880
anti-PROX1	Cell Signaling Technology	14963
anti-SUMO2	Abcam	ab233222
anti-c-RAF	Cell Signaling Technology	9422
anti-SAE1	Abcam	ab185552
anti-MEK1/2	Cell Signaling Technology	9122
anti-UBA2	Abcam	ab185955
anti-UBC9	Abcam	ab75854
anti-SENP1	Abcam	ab108981
anti-SENP2	Abcam	ab124724
anti-SENP3	Abcam	ab124790
anti-SENP5	Abcam	ab58420
anti-SENP6	Abcam	ab77619
anti-SENP7	Sigma-Aldrich	SAB4502484
anti-p-MEK1/2	Cell Signaling Technology	9154
anti-p-c-RAF	Cell Signaling Technology	9427
IHC:		
anti-LYVE-1	Abcam	ab218535

anti-hnRNPA1	Abcam	ab5832
anti-Podoplanin	Abcam	ab236529
anti-Ki67	Sino Biological Inc	ZM-0166
anti-PROX1	Abcam	ab199359
anti-luciferase	Abcam	ab185924
anti-VEGFR3	Abcam	ab51496
anti-CD31	Abcam	ab28364
anti-NRP2	Abcam	ab234821
anti-α-SMA	Abcam	ab7818
anti-CD68	Abcam	ab283654
IF:		
anti-hnRNPA1	Abcam	ab5832
anti-TSG101	Abcam	ab83
anti-CD63	Abcam	ab217345
anti-LYVE1	Cell Signaling Technology	67538S
anti-PKH67	Abcam	ab204951
IP:		
anti-hnRNPA1	Abcam	ab5832
anti-His	Abcam	ab5000
RIP:		
anti-hnRNPA1	Abcam	ab5832
**Secondary antibody:**		
Western Blotting:		
anti-rabbit IgG-HRP	Cell Signaling Technology	7074
anti-mouse IgG-HRP	Cell Signaling Technology	7076
IHC:		
anti-rabbit IgG-HRP	Proteintech	SA00001-2
anti-mouse IgG-HRP	Proteintech	SA00001-1

Supplemental Methods

Plasmid construction and retroviral transduction

Lentivirus infection was used to construct the hnRNPA1 gene stable-overexpressed cell lines. The hnRNPA1 gene was cloned into the pCDH-CMV-MCS-EF1-Puro (Cat#VT1480) lentivirus vector, which was co-transfected with the packaging vectors, psPAX2 (Addgene, Cat# 12260, RRID: Addgene_12260) and pMD2.G (Addgene, Cat#12259, RRID: Addgene_12259) into HEK-293T cells (ATCC, Cat#CCLV-RIE 1018, RRID: CVCL_0063). The lentiviruses were harvested and concentrated from the culture media 72 h after transfection. Subsequently, PANC-1 cells (ATCC, Cat#CRL-1469MET, RRID: CVCL_A4BT) were infected with the lentiviruses and were selected by treatment with puromycin (Sigma Aldrich, MO, USA) for 2 weeks. For cell transfection, siRNA or plasmid, purchased from Genepharma company (Shanghai, China), were transfected using Lipo3000 (Invitrogen, Cat# L3000-015, Waltham, MA, USA) according to the manufacturer's protocol. The cells were plated in 6-well plates at 2×10^5cells/well overnight before transfection. The culture media and the indicated cells were collected after 48h transfection. Subsequently, the culture media from replicate wells of 6-well plate in the same batch and same treatment group were pooled together and subjected to EVs isolation.

IHC analysis

The formalin-fixed and paraffin-embedded tissues obtained from patients with PDAC or the nude mice in the animal experiments were further used for IHC analysis. Briefly, the sections were dewaxed with dimethylbenzene and hydrated in gradient alcohols. Subsequently, the antigens were retrieved using citrate sodium and the sections were blocked in goat serum followed by incubation with primary antibodies at 4°C overnight. After secondary antibodies incubation, the location of the antigens was marked using the DAB substrate (Thermo Fisher Scientific, Cat#34002). The nuclei were counterstained using hematoxylin.

RNA extraction and qRT-PCR assays

Total RNAs from cells, tissues, and EVs were extracted using the TRIzol reagent (Life

Technologies, CA, USA) according to the manufacturer's instructions. RNA concentration was measured with NanoDrop ND-2000 spectrophotometer (Life Technologies, Carlsbad, CA, USA) and then 500 ng of total RNAs were reverse transcribed to cDNA using a Prime ScriptTM RT Master Mix (Takara, Shiga, Japan). qRT-PCR was performed using TBGreen II (Takara, Shiga, Japan) and analyzed on a Roche Light-Cycler system (Roche, Basel, Switzerland). The data of mRNA expression were analyzed using the $2^{-\Delta\Delta CT}$ method and normalized to the expression of *GAPDH* in cells or a synthesized exogenous reference, λ polyA+ RNA from External Standard Kit (Takara, Shiga, Japan) in EVs. The primer sequences are shown in Supplemental Table 6.

Isolation and purification of EVs

The EVs were isolated from the cell culture medium or fresh tissues using differential centrifugation. Cells were cultured in medium supplemented with EV-depleted FBS (obtained from centrifugation at 120,000 × *g* for 16h) for 48h. Fresh tissues were weighted and treated with Collagenase I in culture media at 37℃ for 20 min. The collected cell culture medium and tissue digests were centrifuged at 2,000 × *g* for 10 min to remove the cells and debris, followed by centrifugation at 10,000 × *g* for 30 min to remove the micro vesicles. Finally, the supernatant was further centrifuged at 120,000 × *g* for 70 min and the pellet was washed in PBS by centrifugation at 120,000 × *g* for another 70 min. The pelleted EVs were resuspended in PBS and stored at -80 ℃ for further use. All centrifugations were conducted at 4 ℃. The EVs from serum samples were extracted using the exosome isolation kit (Thermo Fisher Scientific) followed the instructions of the manufacturer. The EVs were quantified by their protein content using BCA Protein Assay Kit (Pierce, Rockford, IL, USA).

Electron microscopy analysis

The characteristics of EVs were identified using transmission electron microscopy (TEM). The isolated EVs were deposited on the grid for 60 min and fixed with 2.5% glutaraldehyde for 10 min. The grids were washed with PBS five times, followed by incubation with uranyl acetate for 5 min. Finally, the grids were washed with PBS, dried, and imaged using a Hitachi transmission electron microscope (Hitachi, Tokyo, Japan).

Nanoparticle tracking analysis (NTA)

The pellet of EVs were diluted in PBS to obtain concentrations up to 2×10^9 particles/ml. The concentration and size of EVs were assessed using Nanosight LM10 (Malvern, Framingham, MA) by recording for 30 s with a 488 nm laser at 25°C. Analysis was performed using the NTA v3.1 software (Malvern, Framingham, MA).

Tube formation assays

Pre-cooled 24 well chambers were coated with growth factor reduced Matrigel (BD Biosciences, San Jose, CA, USA) diluted in ECM (1:2, v/v), and then the chambers were incubated at 37 °C for 30 min. 1×10^5 HLECs cells were seeded in the chambers, followed by treatment of PBS or 10 μg/ml EVs for 12 h, respectively. Finally, the formation of lymphatic vessels was imaged using inverted fluorescence microscopy. The length of the tubes was determined using Image J software (ImageJ, RRID:SCR_003070). Experiments were performed at least three times.

Transwell assays

Cells were harvested and counted after 48 h of transfection. 1×10^5 cells suspended in 100 μl of FBS-free culture medium were seeded into the upper chamber (24-well insert, 8 μm, Corning Costar Corp, NY, USA). PBS or 10 μg/ml EVs were added to the upper chamber. The lower chamber was filled with 700 μl of culture medium containing 10% FBS. The cells were incubated at 5% CO_2 at 37 °C for 18 h and then fixed with methanol for 15 min, followed by staining with 0.1% crystal violet for 15 min. The cells were photographed and five random fields were counted. Experiments were performed at least three times.

Co-immunoprecipitation (Co-IP) assays

Co-IP assays were conducted according to the manufacturer's instructions of the Co-Immunoprecipitation Kit (Pierce, Rockford, IL, USA, Cat#26149) to analyze the post-transcriptional modifications and interacting proteins of hnRNPA1. Briefly, 1×10^7 PDAC

cells were lysed in lysis buffer. The cell lysate was added to an amine-reactive resin pre-coupled with antibodies and the mixture was immunoprecipitated at 4 ℃ overnight. The eluted proteins were further analyzed using mass spectrometry and Western Blotting analysis. Normal rabbit IgG was used as the negative control. The antibodies are list in Supplemental Table 7.

Western Blotting analysis

Total protein was extracted using RIPA lysis buffer supplemented with protease inhibitors. The protein concentration was determined using a BCA Protein Assay Kit (Pierce, Rockford, IL, USA). Proteins were separated by electrophoresis in 10% SDS polyacrylamide gels (SDS–PAGE), and then transferred to a polyvinylidene fluoride membrane. Subsequently, the membrane was blocked with 5% BSA for 1 h at room temperature and then incubated with primary antibodies dissolved in TBST at 4 ℃ overnight. Then, the membrane was incubated with specific HRP-conjugated secondary antibodies for 1 h at room temperature. ECL reagents (Thermo Fisher Scientific, Cat#32109) were used to detect and visualize the immunoreactive proteins. The antibodies are shown in Supplemental Table 7.

Immunofluorescence

Cells were washed with PBS three times, fixed with 4% formaldehyde for 15 min, and permeabilized with 0.1% Triton X-100 (Sigma-Aldrich, Cat#T8787) for 20 min. After blocking with goat serum for 30 min, the cells were incubated with primary antibodies at 4℃ overnight, followed by secondary antibodies in the dark for 1 h. The nuclei were stained with DAPI (Thermo Fisher Scientific, Cat#P36935) for 5 min and the cells were washed with PBS three times before imaging under an LSM710 confocal microscope (Zeiss, Pleasanton, CA,

USA).

Dual-luciferase assays for the promoter and 3'-UTR activity

The promoter and 3'-UTR activities of PROX1 were analyzed by dual-luciferase reporter assays. PROX1-WT, PROX1-mut were inserted downstream of the luciferase gene in psiCHECK2 plasmids while PROX1-promoter was inserted upstream of the luciferase gene in pGL3 plasmids. Cells were seeded in 6-well plates and transfected with the above constructed psiCHECK2 or pGL3 plasmids, followed by treatment with the 10 μg/ml indicated EVs. The empty psiCHECK2 (RRID:C8021) or pGL3 vector (RRID: Addgene_17186) was set as a negative control. The luciferase activities were measured according to the instruction of Dual-Luciferase Reporter Assay System (Promega, Madison, WI, USA). The normalized firefly activity was obtained as the ratio of firefly to Renilla luciferase signals.
